# Study of dense granule proteins in *Eimeria* spp. identifies a limited repertoire with potential as vaccine candidates

**DOI:** 10.3389/fimmu.2025.1605984

**Published:** 2025-07-16

**Authors:** Gonzalo Sanchez-Arsuaga, Damer P. Blake, Fiona M. Tomley, Virginia Marugan-Hernandez

**Affiliations:** Department of Pathobiology and Population Sciences, Royal Veterinary College, Hertfordshire, United Kingdom

**Keywords:** *Eimeria*, dense granule, GRA9, coccidiosis, vaccine, control

## Abstract

*Eimeria* species are obligate intracellular parasites that usually replicate in intestinal cells and can cause the disease coccidiosis. Coccidiosis of livestock and poultry incurs significant production losses and compromises animal welfare, with the greatest impact occurring in chicken production. Control of coccidiosis is challenging, requiring good husbandry and effective drug or live vaccine prophylaxis, and there is demand for more scalable and cost-effective vaccines. Proteins secreted from dense granules (GRAs) play crucial roles in intracellular survival in many apicomplexans, but knowledge of these organelles and their secreted proteins in *Eimeria* is extremely limited. In this study, we observed a reduced GRA protein repertoire encoded in *Eimeria* spp. genomes compared to *Toxoplasma gondii* (23 vs. 71) with just 12 orthologues identified in the *Eimeria tenella* genome. *In silico* analysis of three of these putative dense granule proteins - EtGRA9, EtGRA12a, and EtGRA12b - revealed the presence of signal peptides in EtGRA9 and EtGRA12b, while none was detected in EtGRA12a. Sequence analysis of *E. tenella* field isolates revealed a limited number of polymorphisms in the genes encoding these three proteins, suggesting purifying selection possibly related to their functional importance. In contrast, *E. maxima* showed greater variation, indicating relaxed selective pressure or diversifying selection. When expressed as recombinant proteins all three were recognised by sera from chickens previously infected with *E. tenella*. Their potential role in parasite intracellular niche formation and close interaction with the host cell makes them promising antigens for vaccine development. To evaluate this potential, EtGRA9 was expressed as a recombinant protein and used to vaccinate chickens prior to *E. tenella* challenge. Immunisation with recombinant EtGRA9 reduced parasite load in the caeca by 85.7%, comparable to immunisation with recombinant EtAMA1, an antigen previously shown to confer significant protection.

## 1 Introduction

Coccidiosis in chickens is a severe intestinal disease caused by infection with one or more protozoan parasites from the genus *Eimeria*, phylum Apicomplexa (1), with profound implications for animal well-being and productivity. Coccidiosis has been estimated to cost the global chicken production sector over £10 billion every year (2), compromising chicken weight gain and feed conversion with associated costs for control, including during subclinical infection. As the poultry industry is vital to global food security (3), managing coccidiosis is essential to protect both animal and human health (4, 5).

Since 1948, coccidiosis management in intensive chicken production has primarily relied on anticoccidial drugs administered through feed or water (6). However, their use has raised concerns about selection for drug resistance, environmental and food residues, and inconsistent efficacy across *Eimeria* spp. (7, 8). Live and live-attenuated anticoccidial vaccines provide an alternative for coccidiosis control, inducing robust, long-lasting immunity and closely mimicking natural exposure without driving resistance (9, 10). However, despite efficacy comparable to anticoccidial drugs (6), the adoption of live non-attenuated vaccines has been limited in the broiler industry due to risks of disease transmission in multi-age flocks (11–13), concerns about timely immunity in short-lived broilers (14), and potential impacts on early growth (15). Live attenuated anticoccidial vaccines have overcome many of these challenges, but uptake has been hindered by relatively high vaccine production costs and limited production capacity due to constraints in *in vivo* amplification (16–19).

Recombinant vaccines have long been considered a promising alternative to anticoccidial drugs and live vaccines, with potential benefits for layer and breeding birds and possibly broilers by increasing productivity and reducing drug use. Understanding host-pathogen interactions, such as the role of surface antigens (SAGs) and proteins secreted from micronemes (MICs) in parasite movement and invasion, has aided in the identification of vaccine candidates (20–27). However, despite efforts since the 1980s (28), no recombinant anticoccidial vaccine has reached the market, partly due to challenges in linking immunogenicity with immune protection (29). Several *Eimeria* antigens have been identified as vaccine candidates, including Apical Membrane Antigen 1 (AMA1) (30, 31), Immune Mapped Protein 1 (IMP1) (30, 32), Microneme Protein 3 (MIC3) (23), and others (16). Administering these antigens in the form of recombinant proteins expressed in different systems (e.g. *Escherichia coli)* (33), DNA vaccines (34), or through vectored techniques like transgenic *Eimeria* (24), *Salmonella typhimurium* (35) or *Saccharomyces cerevisiae* (18, 25) has shown promise in reducing parasite replication and intestinal pathology (23, 24, 30). However, further development and a broader selection of vaccine candidates are needed before a recombinant vaccine becomes viable.

Dense granule proteins (GRAs) are a class of proteins that play crucial roles in the survival and development of coccidian parasites. These proteins are usually stored and secreted from dense granules, spherical, membrane-bound organelles in the cytoplasm of many apicomplexan parasites. Upon entry into a host cell, GRAs contribute to the formation of the parasitophorous vacuole membrane and intravacuolar network, which are essential for intracellular survival of the parasite (36). GRAs have been extensively studied in *Toxoplasma gondii* (37) and many *T. gondii* GRAs are immunogenic antigens, eliciting strong immune responses involving T and B cells (38–41). Additionally, several GRAs contain peptides that bind to major histocompatibility complex (MHC) molecules, suggesting they could be targets for cytotoxic T cells (42, 43). Due to their involvement in parasite development, localisation at the host-parasite interface in the parasitophorous vacuole membrane (PVM), and ability to trigger immune responses, GRAs have emerged as promising candidates for vaccines against coccidian infections (42, 43). A small number of potential *Eimeria* GRA proteins have been identified through genomic, transcriptomic (44–48), and proteomic studies (17). This study aimed to explore the *E. tenella* GRA protein repertoire using *in silico* tools and to identify candidates for immunological evaluation. Focusing on the role of these proteins in host-cell niche establishment and their reported immunogenicity in other coccidia (36, 49–51), we also evaluated EtGRA9 as a vaccine antigen against *E. tenella* infection.

## 2 Materials and methods

### 2.1 Selection of vaccine candidates

#### 2.1.1 Identification of GRA orthologues

Genes encoding GRA proteins in *T. gondii* (strain ME49) were identified by reviewing 38 papers from 1989 to 2022. PubMed and Google Scholar were searched using combinations of the keywords *‘Toxoplasma*’, ‘novel’, ‘GRA protein’, ‘TgGRA’, and numerical designations (e.g., ‘GRA1’). Accession numbers obtained from these studies were used to collect protein data, which were subsequently analysed using ToxoDB (52) [(https://toxodb.org/toxo/app)] in January 2022. The default NCBI BLAST tool was employed to identify genes, genomic sequences, ESTs, or PopSet sequences with BLAST similarity to the input sequences. The pairwise overlap ranged from 1.4% to 11.5%, with an E-value threshold of ≤0.001. This analysis was extended to other *T. gondii* strains (GT1, VEG) and *Eimeria* species to identify additional GRA-coding sequences. Additionally, the filters encompassed “genes” and “data sets” to refine the results, while specific fields including “apollo annotations”, “InterPro domains”, “orthologues”, “PDB chains”, “product descriptions”, “PubMed” and “user comments” were selected to filter the outcomes. The proteins EtGRA9, EtGRA12a and EtGRA12b were selected for further investigation, excluding other candidates due to factors such as incomplete or unavailable sequence data, sequence complexity, and low levels of expression.

#### 2.1.2 *In silico* analysis of protein features

In ToxoDB, the ‘protein features and properties’ section was used to investigate essential traits for EtGRA9, EtGRA12a, and EtGRA12b, defining amino acid composition, predicted molecular weight, charge, and the presence of signal peptides (supported by SignalP 6.0 online software), transmembrane domains and glycosylphosphatidylinositol (GPI) anchors. Hydrophobic regions were identified using ProtScale (53) and the Kyte and Doolittle scale (54). Maximum Likelihood (ML), Neighbour-Joining (NJ) and Unweighted Pair Group Method with Arithmetic Mean (UPMGA) phylogenetic trees were generated with 1,000 bootstrap support to compare *E. tenella* proteins with homologous sequences from other parasites. Homologous sequences were identified using BLASTp and recovered from ToxoDB, then aligned and curated using CLC Main Workbench (version 8.1.3; Qiagen, Denmark). ML trees were inferred using MEGA 11 (55) with LG+G (GRA9) or JTT+G (GRA12a and b combined) substitution models. Antigenic determinants were predicted with Kolaskar and Tongaonkar’s method (56), and natively disordered regions were identified using PrDOS (57). Predicted protein structures were generated using I-TASSER (58) and AlphaFold (59, 60) with default parameters, incorporating threading, ab initio modeling, and comparisons with PDB structures. Additionally, B-haplotype epitope binding prediction for EtGRA9 utilised SYFPEITHI software [(www.syfpeithi.de)] (61). Chicken B-F4, B-F12, B-F15, and B-F19 class I molecules were considered, and epitopes were identified based on the amino acid sequence.

#### 2.1.3 PCR

Plasmids and primers were designed using CLC Main Workbench v22 (Qiagen, Hilden, Germany; Toolbox > Molecular Biology Tools > Primers and Probes > Design Primers). PCR amplification was performed using Platinum Taq DNA polymerase (Life Technologies, Paisley, UK). The 25 μl reaction mixture included 1x Taq buffer, 1.5 mM MgCl_2_, 0.2 mM dNTPs (Promega, Chilworth, UK), 0.2 μM of each primer (Sigma-Aldrich, Suffolk, UK), 2 U of Platinum Taq DNA Polymerase, and template DNA, prepared with DNase- and RNase-free water (Fisher Scientific, Loughborough, UK). Thermal cycling, conducted on a SimpliAmp Thermal Cycler (Fisher Scientific), began with an initial denaturation step at 95°C for 1 minute, followed by 30 cycles of denaturation at 95°C for 30 seconds, annealing at a temperature range of 52–58°C (depending on the primer) for 30 seconds, and elongation at 72°C for 1 minute per kilobase of the amplicon. A final extension step at 72°C for 10 minutes completed the protocol. DNA fragment electrophoresis was performed using 0.8–1.5% (w/v) agarose gels. Molecular grade agarose powder (Fisher Scientific) was dissolved in 50 ml of 0.5x tris-borate-ethylenediaminetetraacetic acid (TBE) buffer (Promega).

#### 2.1.4 Validation of annotated ToxoDB sequences and analysis of single nucleotide polymorphisms

Genomic (gDNA) and complementary (cDNA) DNA extracted from sporulated oocysts of the *Eimeria tenella* Houghton reference strain, as previously described (62) were used as templates to amplify *etgra9*, *etgra12a* and *etgra12b* using specific primers (**Supplementary Table 1**). The resulting PCR products were cloned into pGEM T-Easy plasmids (Promega), transformed into *Escherichia coli* Single-Use Competent Cells (JM109) (Promega), and plasmid DNA was extracted using the QIAprep Spin Miniprep Kit (Qiagen), following the manufacturers’ recommended protocols. The extracted plasmids were then sequenced to validate annotations from ToxoDB. gDNA from 18 archive field samples, including *E. tenella* and *Eimeria maxima* from various geographic origins, were used for SNP analysis ((63); **Supplementary Tables 1B, 2**). Genome sequence assemblies from eight *E. tenella* reference strains originating from the UK, US, Japan, and Nigeria were utilised for a broader spatial SNP analysis (64), conducted *in silico* using the local BLAST function. Sequencing data were curated and analysed with CLC Main WorkBench v22.

#### 2.1.5 Expression and purification of recombinant EtGRA proteins

Primers incorporating BamH-I and Not-I sites (**Supplementary Tables 1C, D**) were used to amplify full-length *etgra9* and three sub-section fragments (recEtGRA9-F1, recEtGRA9-F2 and recEtGRA9-F3) (**Supplementary Figure 1**). Coding sequences, excluding the signal peptide, were amplified by PCR from pGEM T-easy plasmids (section 2.1.3), digested by the BamH-I and Not-I restriction enzymes (NEB) and cloned into the pET-32b vector (previously linearised with the same restriction enzymes; Novagen) using T4 ligase (Promega). The recombinant constructs were then transformed into competent cells and plasmid preparations were carried out as described previously (section 2.1.4). The clones were sequenced by GATC Biotech Services (Eurofins, Konstanz, Germany) using T7 and SP6 primers (present in the pET-32b plasmid).

For *etgra12a* and *etgra12b*, primers were designed with the NEBuilder Assembly tool (NEB, Hitchin, UK) (excluding signal peptides), and sequences were amplified by PCR and cloned into pET-32b (previously linearised with BamH-I HF and Not-I HF restriction enzymes (NEB)) using NEBuilder HiFi and Gibson Assembly (NEB). Clones were sequenced using T7 promoter and SP6 terminal primers.

Recombinant proteins were expressed in *E. coli* BL-21 (DE3) pLysS (Novagen) cultivated in LB medium with ampicillin (100µM) (Sigma-Aldrich) and induced with 1 mM IPTG (Sigma-Aldrich) when cultures reached an optical density (OD600) of 0.6. After 4 hours of induction, bacterial pellets were collected and lysed using BugBuster^®^ reagent (Millipore) or in-house lysis buffer, following the same method as Marugan-Hernandez et al. (62). The lysate was sonicated at 20 kHz for 10 minutes in 30-second bursts, then centrifuged at 16,000 x g for 15 minutes at 4°C, with the resulting lysate and pellets stored at -20°C. Sodium dodecyl-sulphate polyacrylamide gel electrophoresis (SDS-PAGE; section 2.1.6) was used to assess the presence of induced proteins. The presence of recEtGRAs, tagged with five histidines (His-tag) at the C-terminus, was validated by immunoblot using anti-His-tag antibodies (Invitrogen). When recGRAs were contained in inclusion bodies (insoluble fraction), these were cleaned in a PBS pH 7.4, 0.5% Triton X-100 solution and solubilised in a PBS pH 7.4, 8M urea, 40 mM imidazole solution. Recombinant proteins were purified from the specific fraction using HisTrapTM HP columns (Cytiva) and the AKTA Start system (Cytiva).

#### 2.1.6 SDS-PAGE and immunoblotting

Depending on the experimental procedure, proteins were electrophoresed at 200 V for 40 minutes using a PS600–600 Volt Power Supply (Hoefer) on either single-lane or 10-well NuPAGE Bis-Tris gels (Invitrogen). Gels were then processed using an XCell SureLock Mini-Cell system (Invitrogen), followed by the transfer of proteins onto PVDF membranes (Cytiva Amersham Hybond). Membranes were incubated with sera at varying dilutions of commercial antibodies for 1 hour. After three 5-minute washes with TBS-Tween 0.05%, membranes were incubated with secondary antibodies for 1 hour. After an additional round of washes, detection was carried out by chemiluminescence with Immobilion Crescendo Western HRP Substrate (Millipore) or chromogenic visualisation with 1-Step TMB-Blotting Substrate Solution (Thermo Fisher Scientific). In the case of chemiluminescence, bands were visualised and captured using UV light with a G BOX imaging system and GeneSnap 7.12 software.

#### 2.1.7 Evaluation of recombinant EtGRA proteins for immune recognition

To evaluate immune recognition by infected chickens, recombinant proteins (recEtGRA9, recEtGRA9 fractions, recEtGRA12a, recEtGRA12b and recEtAMA1 (24) were subject to immunoblot assays against sera from uninfected (naïve) or *E. tenella* infected chickens. In brief, APHA (Lohmann Valo) chickens received a single oral inoculation of 1,000 sporulated oocysts of the *E. tenella* Houghton reference strain at three weeks of age. Blood was collected for serum preparation 7 days post-infection. The collection was carried out by centrifuging the blood at 2,000 × g for 12 minutes at 4°C to obtain the serum. Six micrograms of each recombinant protein were electrophoresed and after immunoblotting were incubated with chicken sera diluted 1:100 (naïve) or 1:10,000 (infected). Detection was carried out with HRP-conjugated secondary antibodies (rabbit anti-chicken IgY (H+L)) (Invitrogen) at a 1:10,000 dilution, followed by chemiluminescent or chromogenic development.

### 2.2 Maintenance of parasites

To amplify *E. tenella* Houghton reference strain, 15 mixed-sex four-week-old specific pathogen-free Lohmann Valo chickens accommodated in wire-floored cages were infected with 4,000 oocysts (65). After one week, birds were slaughtered, and oocysts were collected from the caeca, sporulated, and purified (66) for use in immunisation and challenge within two months, as well as a source for gDNA and cDNA (sections 2.1.4 and 2.1.5).

### 2.3 Assessing vaccine potential of recEtGRA9 against chicken coccidiosis

#### 2.3.1 Animals and parasites

For the vaccination study, 48 female Hy-line Brown layer chickens were sourced from Hy-line UK Ltd on day 0 and arrived already vaccinated against Marek’s disease. All chickens were provided with commercial organic starter feed (anticoccidial-free) and water *ad libitum* and were housed in ammonia-fumigated wire-floored cages.

#### 2.3.2 Experimental design

Chickens were caged in six groups of eight animals and acclimatised for one week (**Table 1**). After wing-tagging for individual identification, groups 1 and 2 served as uninfected and infected controls and did not receive immunisations. Groups 3 to 5 received three immunisations, which were administered subcutaneously at two different points in the interscapular region, starting at one week of age (day 7) with one-week intervals between each dose. Group 3 received adjuvant only, with TiterMax Gold Adjuvant (100 µl/dose; Sigma) for the first two doses (days 7 and 14) and Incomplete Freund’s Adjuvant (100 µl/dose; Sigma) for the third (day 21). Groups 4 and 5 received 50 µg of recEtGRA9 or recEtAMA1 per dose per bird, respectively, with the corresponding adjuvant described for group 3. Group 6 was orally vaccinated with 100 *E. tenella* Houghton sporulated oocysts in 1 ml of distilled water at 2 weeks (day 14) and 300 oocysts at 3 weeks (day 21) to simulate natural parasitic recycling.

**Table 1:** Experimental design and group distribution for the vaccination trial.

Group (N/group)	Treatment	Dose	Route	*E. tenella* challenge
G1 (8)	Unvaccinated, unchallenged control	–	–	No
G2 (8)	Unvaccinated, challenged control	–	–	Yes (300 oocyst/bird)
G3 (8)	Adjuvant control	–	Subcutaneous	
G4 (8)	recEtGRA9	50 ug/bird		
G5 (7)	recEtAMA1			
G6 (6)	*E. tenella* oocysts	100 oocyst/bird 300 oocyst/bird	Oral	

One day before *E. tenella* challenge (day 34), blood was collected from the brachial vein of each chicken to assess antibody responses. Two weeks after the final immunisation (day 35), groups 2 to 6 were challenged with 300 sporulated *E. tenella* Houghton oocysts in 1 ml of distilled water. Chickens were sacrificed 5 days after the challenge (day 40) by cervical dislocation. Blood was collected from the jugular vein immediately post-mortem, and sera were extracted by centrifugation as described previously (section 2.1.7). Caeca were collected and pathological lesions were scored in the right/left caeca according to Johnson and Reid (67), then both caeca were snap frozen in dry ice. Body weights were recorded at 7, 14, 21, 35 and 40 days.

#### 2.3.3 DNA extraction and parasite quantitation by quantitative PCR

Genomic DNA (gDNA) was extracted from thawed caecal tissues after removing faecal contents, slicing and homogenising with RLT Plus buffer and a TissueRuptor II (Qiagen). The homogenate was processed using an AllPrep DNA/RNA Mini Kit as recommended by the manufacturer (Qiagen). gDNA quality and concentration were assessed with a Nanodrop DeNovix DS-11 Series. Quantitative real-time PCR (qPCR) was performed as described previously (68) to assess *E. tenella* genome copy number in the caeca using a CFX96 Touch^®^ Real-Time PCR Detection System (Bio-Rad). Primers for the *E. tenella* RAPD-SCAR marker Tn-E03–116 and chicken tata-binding protein (**Supplementary Table 1E**) were used. Standard curves were generated for absolute quantification using plasmids pGEM-EtSCAR and pGEM-TBP, with 10-fold serial dilutions from 10^7^ to 10^1^ copies, as described previously (69).

Analysis of qPCR data was conducted using CFX Maestro Software 2.3 (Bio-Rad). The number of genomes from the chicken (TBP) and the *E. tenella* (SCAR) was compared to the plasmid standard series. Triplicate data (parasite genome copy number) was averaged for each sample and standardised by comparison with the host genome (chicken) copy number as a ratio of *E. tenella*/chicken genomes. Individual data was excluded if the standard deviation from the triplicate analysis exceeded 0.5.

#### 2.3.4 Immunoblots for pre-challenge sera assessment

Sera collected post-vaccination but prior to the *E. tenella* challenge were tested against recEtGRA9, recEtAMA1, and a lysate of 20 million *E. tenella* sporozoites, obtained by treating the sporozoites using ice-cold PBS buffer (pH 7.4) containing imidazole (40 mM), glycerol (5%), and Triton X-100 (0.5%). This was followed by a 5-minute incubation and sonication to enhance protein yield. Each recombinant protein (6 μg) was processed via SDS-PAGE and transferred to PVDF membranes as described in section 2.1.6. Sera were diluted 1:100 for negative controls and 1:10,000 for immunised chickens, followed by incubation using a Mini-PROTEAN II apparatus (BioRad). Membranes were then incubated with a 1:10,000 dilution of secondary antibody (polyclonal anti-chicken IgY-peroxidase, Invitrogen), and developed using chemiluminescent detection, with bands visualised as outlined in section 2.1.6.

#### 2.3.5 Statistical analysis

Statistical analyses were conducted with GraphPad Prism 10 using one-way ANOVA for weight gain (followed by Tukey’s test for *post-hoc* comparisons), paired t-test for caecal parasite load, and Kruskal-Wallis test for lesion scores.

### 2.4 Ethics statement

All work with chickens complied with the Animals (Scientific Procedures) Act 1986 and was performed under the UK Home Office Licence, with approval from the Royal Veterinary College Animal Welfare Ethical Review Body (AWERB).

## 3 Results

### 3.1 Comparative analysis with *T. gondii* identified 23 *Eimeria* GRA protein orthologues

Analysis of *Eimeria* genomes identified a limited number of genes predicted to encode proteins with similarity to *T. gondii* GRA proteins (TgGRAs). A total of 71 GRA encoding genes were identified in the *T. gondii* GT1, ME49 and/or VEG genome sequence assemblies (**Supplementary Table 3**). Of these 71, orthologues were detected for 23 in at least one *Eimeria* species (**Supplementary Table 3**). Two more *Eimeria* GRA proteins were identified by ‘name’ searching in ToxoDB (putative GRA10bis and GRA11bis); however, they were not found to be orthologues to *T. gondii* proteins. For *E. tenella*, genes encoding 12 putative orthologues of *T. gondii* GRA proteins were identified (EtGRA9, EtGRA10, EtGRA11, EtGRA12a, EtGRA12b, EtGRA38, EtGRA44, EtGRA52, EtGRA53, EtGRA61, EtGRA63, EtGRA66) (**Supplementary Table 3**). Among these, the extent of similarity with their corresponding orthologous *T. gondii* proteins varied from 19% for EtGRA52 to 42.34% for EtGRA66, based on the full length of each protein (**Supplementary Table 3**). The lowest similarity values were found for TgGRAs associated with cyst walls (not a feature of the *Eimeria* lifecycle), namely TgGRA52 and TgGRA53. Conversely, the highest similarity percentages were observed for proteins targeted to the PVM in *T. gondii*, such as TgGRA44 and TgGRA66.

### 3.2 GRA9, GRA12a and GRA12b demonstrated distinct lineages across *Toxoplasma*, *Cyclospora*, and *Eimeria*

EtGRA9, EtGRA12a and EtGRA12b were selected for further analysis. EtGRA9 and EtGRA12a have previously shown abundant expression as they were identified in multiple proteomic studies (17, 48). Moreover, EtGRA9 was shown to be secreted from sporozoites in a Cas9/CRISPR tagging experiment (45). EtGRA12b expression was not detected in these studies but was included here due to the similarity in amino acid sequence length and intron/exon structure with EtGRA12a, plus the availability of well-annotated sequences.

**Figure 1:**
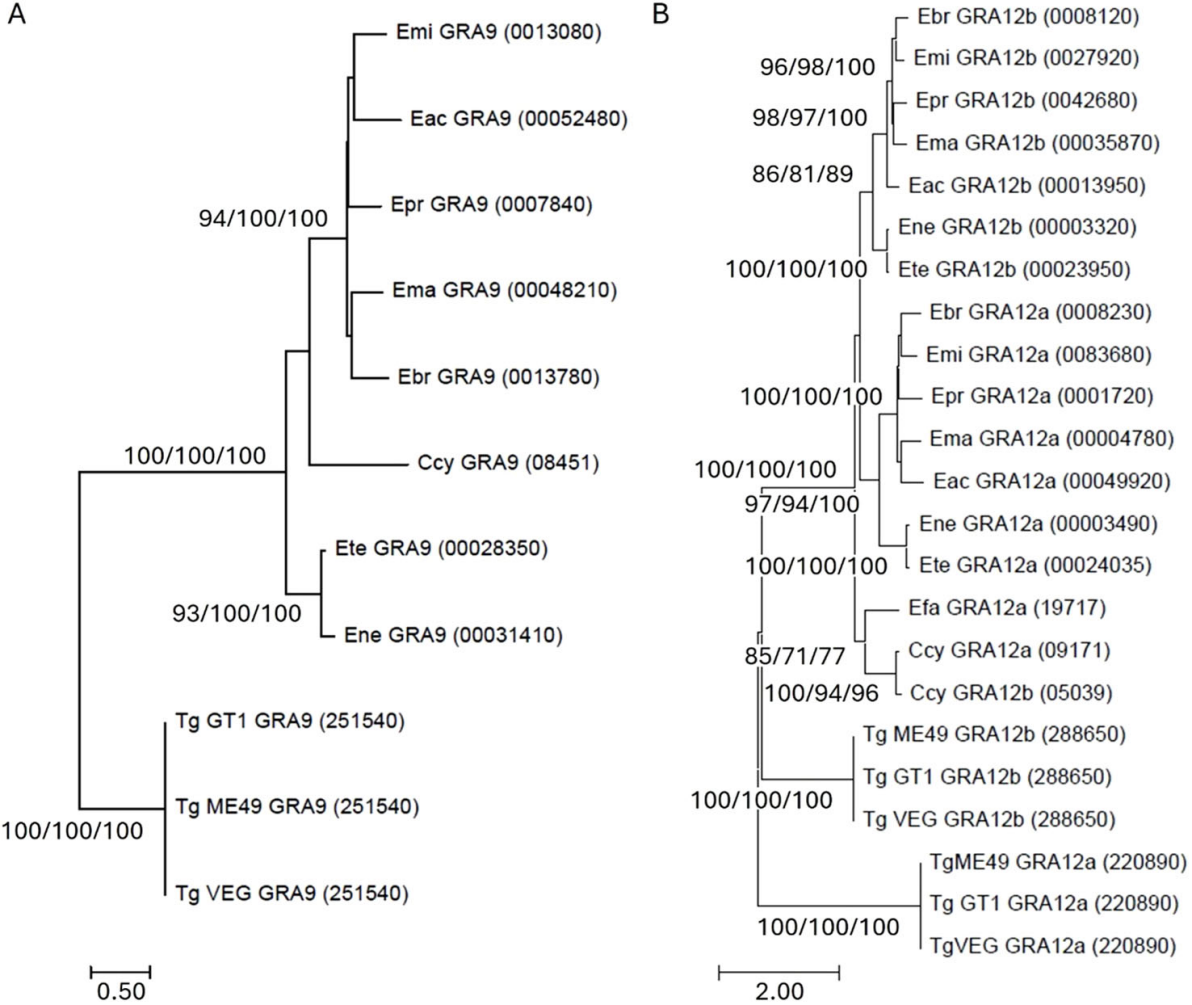
Optimal Maximum Likelihood (ML) tree for **(A)** *Eimeria* spp. GRA9 inferred from a 361 amino acid alignment using LG + gamma, and **(B)** *Eimeria* spp. GRA12a and b from a 489 amino acid alignment using JTT + gamma, compared to *Toxoplasma gondii* (Tg) and *Cyclospora cayetanensis* (Ccy) orthologues. The percentage of 1,000 bootstrap iterations presenting with this topology in more >70% of examples is shown for ML/Neighbour-Joining (NJ)/Unweighted Pair Group Method with Arithmetic Mean (UPMGA) methods. Eac, *Eimeria acervulina*; Ebr, *Eimeria brunetti*; Efa, *Eimeria falciformis*; Ema, *Eimeria maxima*; Emi, *Eimeria mitis*; Ene, *Eimeria necatrix*; Ete, *Eimeria tenella*.

Phylogenetic analysis revealed the presence of four distinct clades that illustrate the evolutionary relationships within GRA9 among *T. gondii*, *Cyclospora cayetanensis* and *Eimeria* (**Figure 1**). The first clade comprised TgGRA9, reinforcing a lineage-specific to this species as the only representative in the analysis external to the Eimeriidae family. The second clade illustrated a close association between *E. necatrix* and *E. tenella*. *C. cayetanensis* GRA9 (CyGRA9) resolved in a clade on its own, closely related to the fourth clade that encompassed *Eimeria brunetti, Eimeria maxima, Eimeria praecox, Eimeria acervulina* and *Eimeria mitis*. The two *Eimeria* clades exhibited a higher degree of similarity to each other compared to their similarity with *T. gondii*. Furthermore, both *Eimeria* clades maintained a closer resemblance to the CyGRA9 homologue in *C. cayetanensis* than to *T. gondii*.

Analysing GRA12a and GRA12b protein sequences together placed all GRA12 sequences from *Eimeria* that infect chickens in distinct sub-clades for a and b sequence types (**Figure 1**). Comparison with Eimeriidae that infect mammals revealed a distinct clade for *C. cayetanensis* GRA12a and b sequences, most closely related to GRA12a from *Eimeria falciformis* – a species that specifically infects the house mouse (*Mus musculus*). GRA12a and b sequences from *T. gondii* were more distantly related.

### 3.3 Protein profiling illustrated the presence of signal peptides, balanced hydrophobicity and antigenic determinants, and low degree of disordered regions

EtGRA9 was predicted to comprise 271 amino acids with an N-terminal signal peptide composed of 23 amino acids (**Supplementary Table 4**). EtGRA12a includes 407 amino acids and contains an N-terminal region spanning 26 amino acids, initially presumed to function as a signal peptide (**Supplementary Table 4**). However, unlike classical secretory signal peptides, this region includes three highly polar arginine residues, reminiscent of twin-arginine (RRR) translocated signal peptides. Specifically, when the protein is truncated to remove the RRR sequence, the prediction score drops below 0.5, suggesting that EtGRA12a does not possess a functional signal peptide (**Supplementary Figure 2**). Additionally, while a transmembrane (TM) domain feature was identified, its hydrophobic nature is not strongly predicted. EtGRA12b encoded a protein comprising 395 amino acids with an N-terminal signal peptide but, unlike EtGRA12a, it lacked a transmembrane domain. No signal for the addition of a glycosylphosphatidylinositol anchor was detected (**Supplementary Table 4**).

The hydrophobicity scores of all proteins exhibited a range of -3 to +3, reflecting a balanced distribution of hydrophobic and non-hydrophobic regions in a near 1:1 ratio, resulting in overall neutral profiles. A similarity in hydrophobicity patterns was observed among all the proteins, with the highest degree of resemblance observed between the EtGRA12a and EtGRA12b orthologues, where the patterns were nearly identical despite sequence divergence (**Supplementary Figure 3**).

Consistent antigenicity patterns were observed across all three EtGRAs, with average antigenic propensities ranging from 1.0205 to 1.0376 (> 1.00 are considered potential antigenic determinants), suggesting the presence of regions likely to be surface-exposed within the molecule (**Supplementary Figure 4**). Moreover, the assessment of the three candidate EtGRAs predicted the presence of 11 to 17 antigenic determinants (**Supplementary Table 5**). The expressed recombinant subfragments from EtGRA9 contained varying numbers of complete antigenic determinants: two in Fragment 1, two in Fragment 2, and four in Fragment 3.

Disordered regions enable flexible interactions, potentially influencing host-pathogen dynamics, immune evasion, and pathogenicity while serving as post-translational modification sites and immune targets. Predicted disordered regions were analysed, considering residues with scores above the 0.3 threshold as disordered. EtGRA9 exhibited a protein structure without prominent disordered regions. However, EtGRA12a and EtGRA12b displayed distinctive patterns, with two peaks at the terminal region, indicating the presence of disordered regions with propensity values around 0.6 (**Supplementary Figure 5**). Similar patterns were observed in their *T. gondii* orthologues, highlighting terminal regions as areas displaying disorder (**Supplementary Figure 6**).

**Figure 2:**
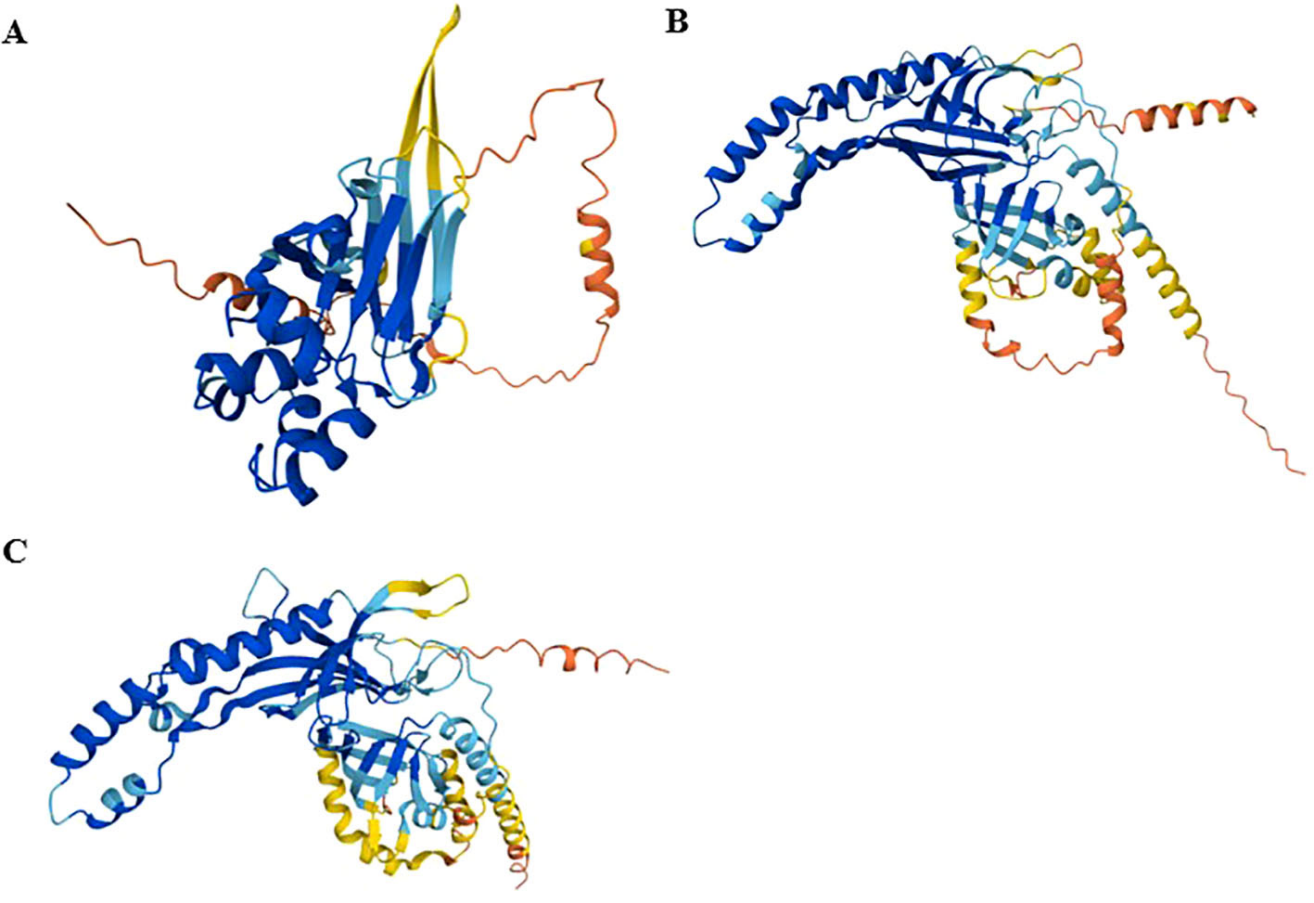
Tertiary 3D protein structures of EtGRA9 **(A)**, EtGRA12a **(B)**, and EtGRA12b **(C)**, predicted using AlphaFold. Colours represent per-residue confidence levels (pLDDT): very high (pLDDT > 90) in dark blue, high (90 > pLDDT > 70) in light blue, low (70 > pLDDT > 50) in yellow, and very low (pLDDT < 50) in orange. Regions with pLDDT below 50 may be unstructured in isolation.

The predicted 3D conformation of EtGRA9, generated using AlphaFold (**Figure 2**), suggests a compact globular structure with a distended conformation predominantly composed of alpha-helices. The model features multiple secondary structural elements, including intricately folded alpha-helices and beta-strands forming a defined hydrophobic core. The structure comprises two distinct domains: a central core dominated by three antiparallel β-sheets in a sandwich-like arrangement, flanked by N- and C-terminal regions enriched in α-helices. I-TASSER further identifies three dispersed binding sites within the EtGRA9 protein sequence (**Supplementary Figure 7A**).

Structural predictions for EtGRA12a and EtGRA12b from AlphaFold suggest a propensity to adopt coiled-coil motifs in their 3D conformations, characterised by repeating alpha-helical segments (**Figure 2**). Notably, I-TASSER predictions indicated a high concentration of potential ligand binding sites clustered in the core regions of these proteins (**Supplementary Figures 7B, C**). Additionally, analysis of EtGRA12b reveals the presence of domains resembling those of an inositol-3-phosphate synthase enzyme, including a predicted active site (**Supplementary Figure 7D**).

Further EtGRA9 protein sequence analysis using SYFPEITHI software predicted six chicken T-cell epitope sites (**Table 2**). These epitopes target four distinct chicken B-haplotypes (B-F12, B-F15, B-F19 and B-F4) encoding the MHC-I molecule. Four identified epitope sites were specific for the B-F12 haplotype, while a single epitope is recognised for both B-F19 and B-F4. Notably, no epitope for the B-F15 molecule was found.

**Table 2:** Summary of the chicken T-cell epitopes predicted within etgra9 using SYFPEITHI software.

MHC-I molecule	Anchors (position)	Present	Number	Amino acid position	Reference
Chicken B-F12	V, I (5) V (9)	YES	4	V(112)-V (116) V (150)-V(154) I (206)-V(210) V(265)-V(269)	(70)
Chicken B-F15	R (2) Y (8)	NO	0	–	
Chicken B-F19	R (2) Y, P, L, F (8)	YES	1	R(29) - P(35)	
Chicken B-F4	D, E (2) D, E (5) E (8)	YES	1	E(135) - E(138)- E (141)	

### 3.4 Sequence variability was low for *gra9, gra12a* and *gra12b* in *E. tenella* compared to *E. maxima*

The predicted genomic loci in ToxoDB for *etgra9, etgra12a* and *etgra12b* contain a total of 1,005, 1,400 and 1,305 base pairs, spanning three, two and two exons, respectively. *etgra9* and *etgra12b* sequences amplified from the *E. tenella* Houghton strain in this study exhibited a perfect match of 100% sequence identity with the predictions derived from the Houghton reference strain in the database. However, in the case of *etgra12a*, a non-synonymous polymorphism was detected (coding position 12 (L(12)F).

Low levels of sequence variation were detected when DNA from field isolates was used for amplification and sequencing of *etgra9, etgra12a* and *etgra12b* (**Table 3**), revealing one synonymous SNP in *etgra9* (in field sample 23.3, Poland; **Supplementary Table 2**), one non-synonymous SNP in *etgra12a* (present in Houghton and 25.2-New Zealand; **Supplementary Table 2**), and one non-synonymous SNP in *etgra12b* (found in Houghton, Wisconsin, Nippon, and 25.2-New Zealand; **Supplementary Table 2**). In contrast, *E. maxima* exhibited significantly higher polymorphism counts, with *emgra9* showing 13 SNPs, *emgra12a* two SNPs and *emgra12b* three SNPs. These polymorphic sites in *E. maxima* were present in multiple samples, indicating genuine genetic diversity. Specifically, *emgra9* contained 13 polymorphic sites, including eight coding and five non-coding polymorphisms. Among the coding polymorphisms, four were synonymous and four were non-synonymous. *emgra12a* displayed two polymorphic sites within coding regions, with one being synonymous and the other non-synonymous. *emgra12b* had three polymorphic sites: two coding polymorphisms (both non-synonymous) and one non-coding polymorphism. Nucleotide diversity (Pi), a metric for assessing polymorphism within a population, was substantially higher in *E. maxima* than in *E. tenella* (**Table 3**). The dN/dS ratios suggest different selection pressures on the genes. For *etgra12a*, *etgra12b*, and *emgra12b*, the ratios indicate positive selection, where non-synonymous mutations are favoured. For *emgra9* and *emgra12a*, the ratios suggest neutral evolution, where synonymous and non-synonymous mutations occur at similar rates. Finally, for *etgra9*, the ratio indicates purifying selection, where synonymous mutations are favoured (**Table 3**).

**Table 3:** Genetic variation and SNP analysis across sampled species and loci.

	*etgra9*	*etgra12a*	*etgra12b*	*emgra9*	*emgra12a*	*emgra 12b*
No. sequences analysed	15	16	14	11	8	10
No. countries represented	9	9	8	7	5	7
No. sites (base pairs)	1005	1400	1305	1121	1311	1019
No. polymorphic sites	1	1	1	13	2	3
Coding polymorphisms	1	1	1	8	2	2
Non-coding polymorphisms	0	0	0	5	0	1
Synonymous polymorphisms	1	0	0	4	1	0
Non-synonymous polymorphisms	0	1	1	4	1	2
No. haplotypes	2	2	2	10	2	3
Haplotype diversity	0.133	0.233	0.440	0.982	0.429	0.511
Variance of haplotype diversity	0.01261	0.01578	0.01254	0.00215	0.02846	0.02700
Standard deviation of haplotype diversity	0.112	0.126	0.112	0.046	0.169	0.164
Nucleotide diversity (Pi)	0.00013	0.00017	0.00034	0.00563	0.00069	0.00090
Adjusted dN/dS +1	0.5	2	2	1	1	3
Tajima’s (D)	-1.15	-0.44	0.84	0.41	0.41	-1.03

### 3.5 Recombinant EtGRA9, EtGRA12A and EtGRA123B were recognised by sera from chickens previously infected with *E. tenella*

**Figure 3:**
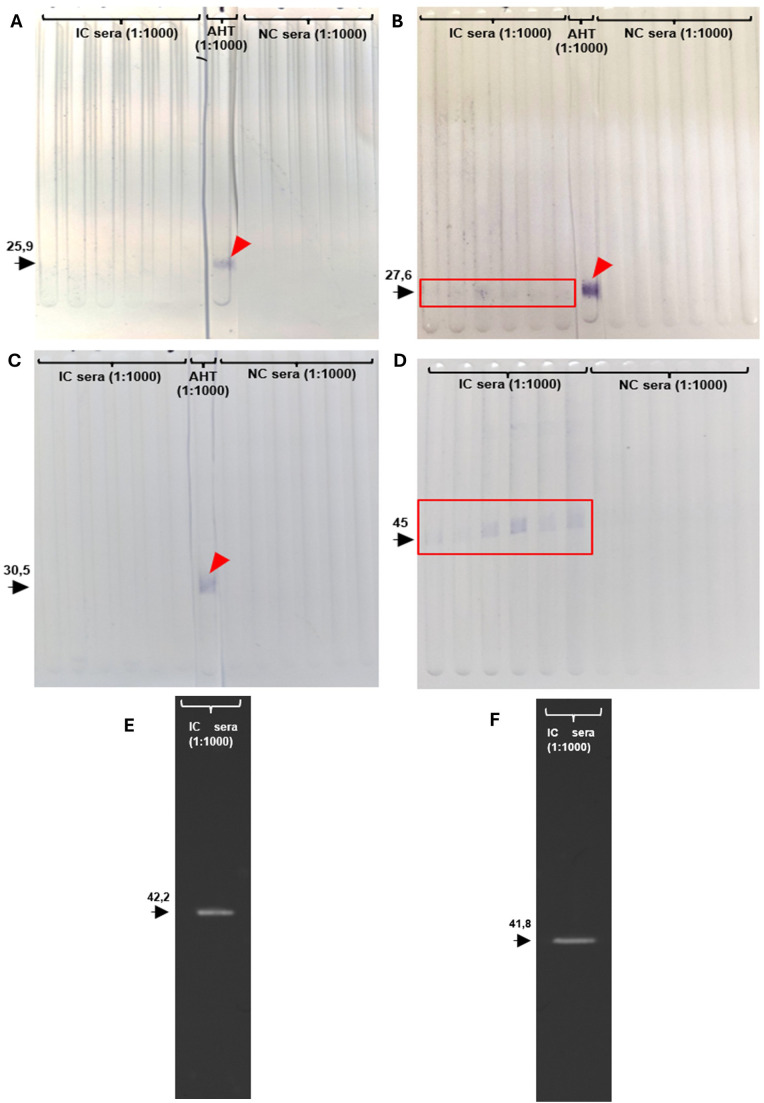
Immunoblots showing recombinant protein recognition. **(A)** recEtGRA9 fragment 1; **(B)** recEtGRA9 fragment 2; **(C)** recEtGRA9 fragment 3; **(D)** recEtGRA9; **(E)** recEtGRA12a; **(F)** recEtGRA12b. Black arrowheads indicate expected recombinant sizes, with molecular weights (kDa) above. Positive recognition is marked with red squares; red arrowheads show recognition by the anti-His tag antibody. Only recEtGRA9 fragment 2 **(B)**, recEtGRA9 **(D)**, recEtGRA12a **(E)** and recEtGRA12b **(F)** exhibited recognition by sera from infected chickens. IC, infected chicken; AHT, anti-His tag antibody; NC, naïve chicken. **(A–D)** represent immunoblots visualised using chromogenic techniques, while **(E, F)** were analysed by chemiluminescence.

Fractions of recEtGRA9 (recEtGRA9-F1, recEtGRA9-F2 and recEtGRA9-F3), recEtGRA9, EtGRA12a, EtGRA12b and recEtAMA1 (all lacking signal peptide) were successfully expressed in *E. coli* and purified as soluble protein (**Supplementary Figure 8**). Sera obtained from chickens previously infected with *E. tenella* consistently recognised full-length EtGRA9 (**Figure 3**), but only recEtGRA9-F2 showed some degree of recognition when the three distinct fragments were tested. EtGRA12a and EtGRA12b also showed clear recognition when incubated with sera from infected chickens (**Figure 3**).

### 3.6 Immunisation of chickens with recEtGRA9 enhanced weight gain following challenge

**Figure 4:**
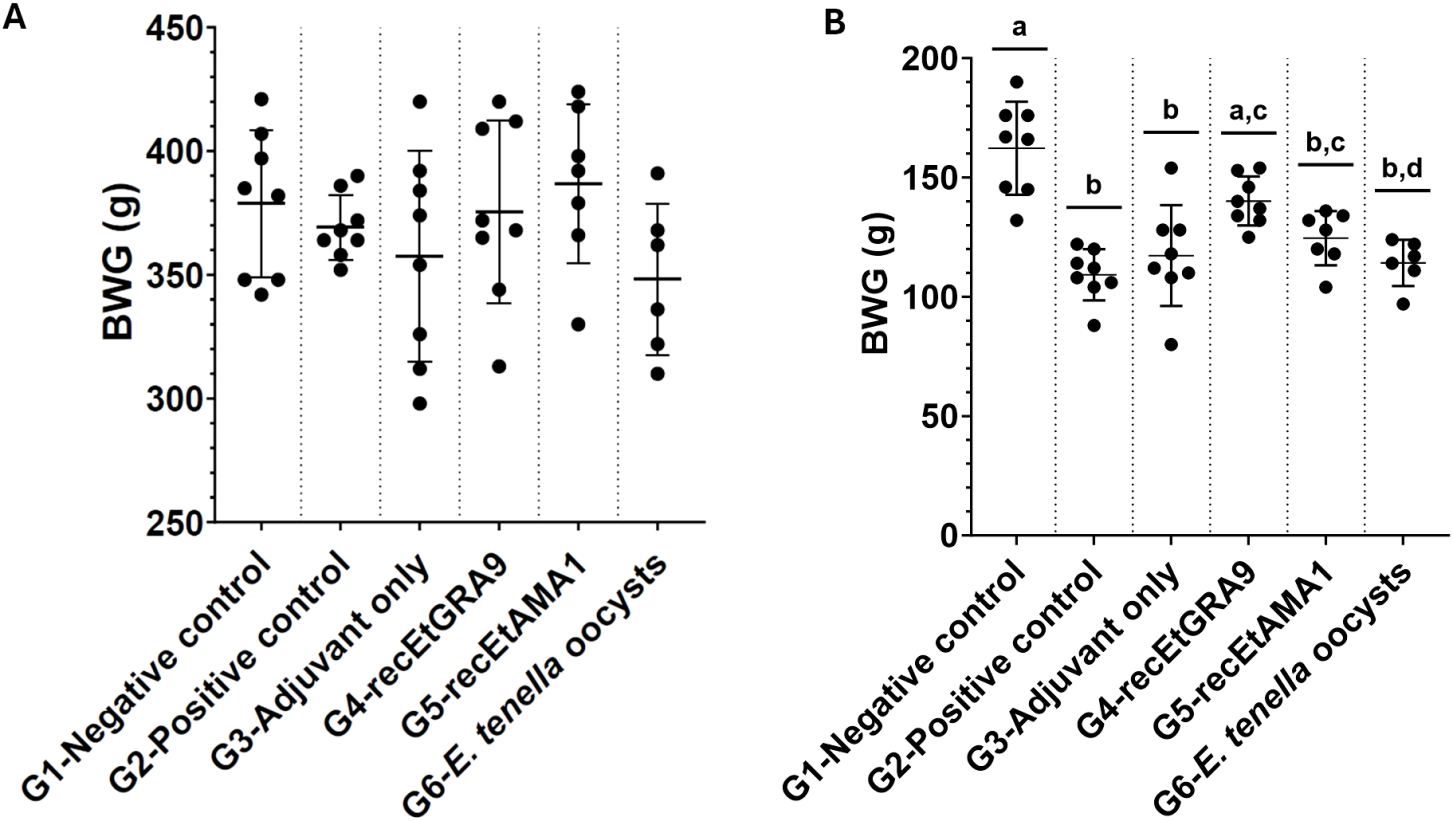
Body weight gain in grams of Hy-line Brown layer chickens, measured **(A)** before the challenge (days 7–35) and **(B)** after the challenge (days 35–40). Experimental groups are shown on the x-axis, and body weight gain is shown on the y-axis. Each dot represents an individual chicken (n=6–8 per group). In **(A)** no significant differences were observed between groups (p > 0.05; One-way ANOVA, Tukey multiple comparison correction). Groups marked with different letters were significantly different (P < 0.05).

The ability of recEtGRA9 to confer immunoprotection against coccidiosis in chickens was evaluated through an *in vivo* trial including a group vaccinated with recEtGRA9 and several controls (**Table 1**). No differences in weight gain were observed between groups before the challenge (**Figure 4A**); however, significant differences were observed after the challenge between unvaccinated unchallenged (negative control) and unvaccinated challenged (positive control) groups (G1 and G2 in **Figure 4B** and **Supplementary Table 6**). A significant improvement in weight gain after challenge was found in the group vaccinated with recEtGRA9 (G4) compared with positive control (G2) and adjuvant-only (G3) group (p>0.001) (One-Way ANOVA and Tukey’s test); **Figure 4B** and **Supplementary Table 6**); weight gain for the recEtGRA9 vaccinated group (G4) increased by 28.3% compared to the positive control group (G2) (**Supplementary Table 6**). Groups immunised with recEtAMA1 and *E. tenella* oocysts (G5 and G6) did not exhibit statistically significant improvement over the positive control.

### 3.7 Specific antibody responses were detected after immunisation with recEtGRA9, recAMA1 and *E. tenella* oocysts

**Figure 5:**
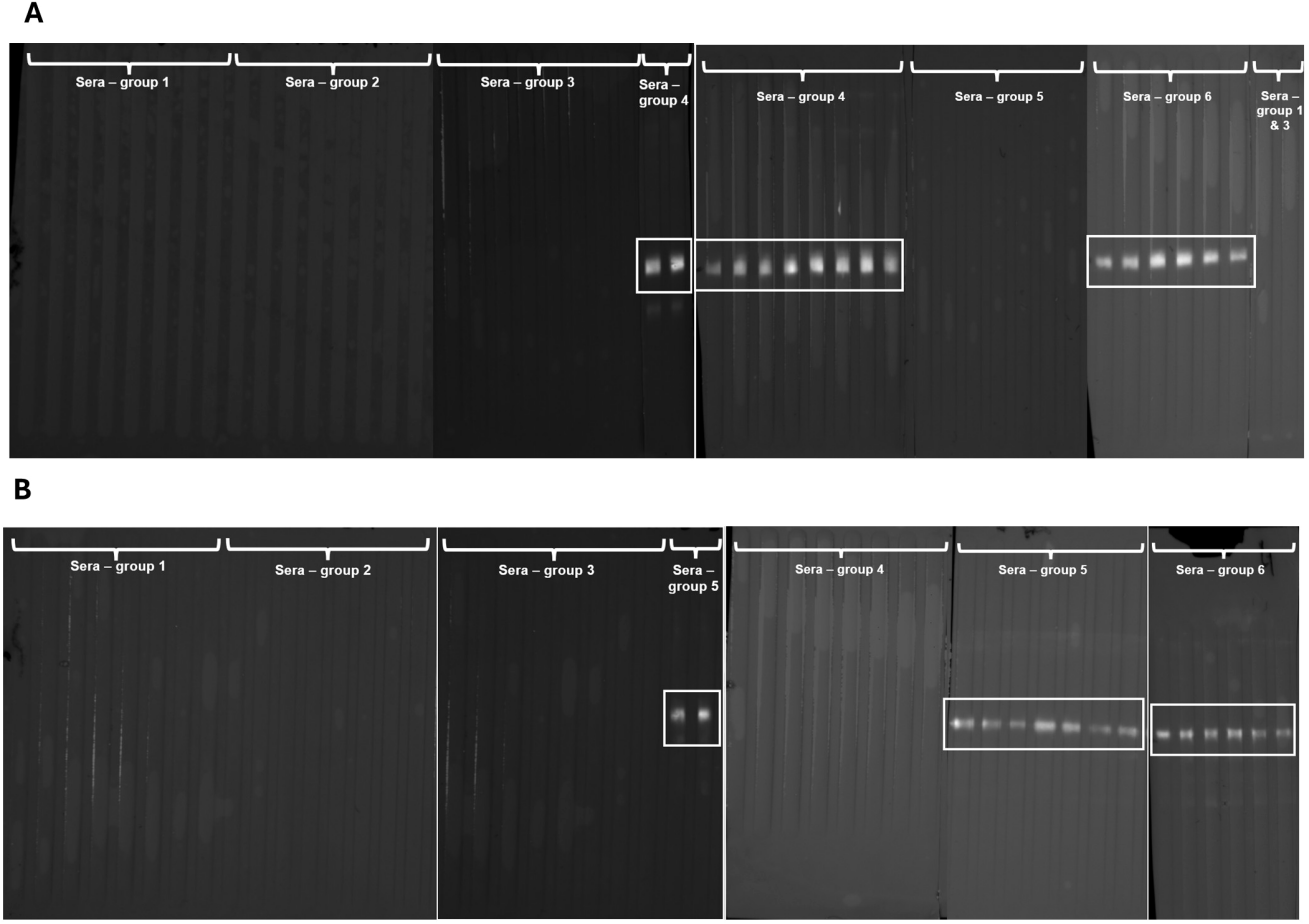
Immunoblots of recEtGRA9 **(A)** and recEtAMA1 **(B)** evaluated against chicken sera from the different experimental groups used in the efficacy study. Group 1: sera from negative control; Group 2: positive control; Group 3: adjuvant only; Group 4: chickens immunised with recEtGRA9; Group 5: chickens immunised with recEtAMA1; Group 6: chickens immunised with *E. tenella* Wis oocysts (**Table 1**). Antibody-antigen recognition is highlighted by a white square. This was observed exclusively in G4 and G6 against recEtGRA9 and G5 and G6 against recEtAMA1.

Sera from the control groups (G1-G3) did not show specific recognition against recEtGRA9 (**Figure 5A**) or recEtAMA1 (**Figure 5B**) by immunoblot. Sera from chickens vaccinated with recEtGRA9 (G4) specifically recognised recEtGRA9 (**Figure 5A**) and failed to recognise recEtAMA1 (**Figure 5B**). Likewise, sera from chickens vaccinated with recEtAMA1 (G5), specifically recognised recEtAMA1 (**Figure 5B**) and did not recognise recEtGRA9 (**Figure 5A**). Sera from chickens vaccinated with oocysts from *E. tenella* (G6) recognised both recombinant proteins, evidencing the presence of specific antibodies generated by chickens against native EtGRA9 and EtAMA1 (**Figures 5A, B**).

### 3.8 Caecal lesions were only reduced in chickens previously exposed to *E. tenella* oocysts

**Figure 6:**
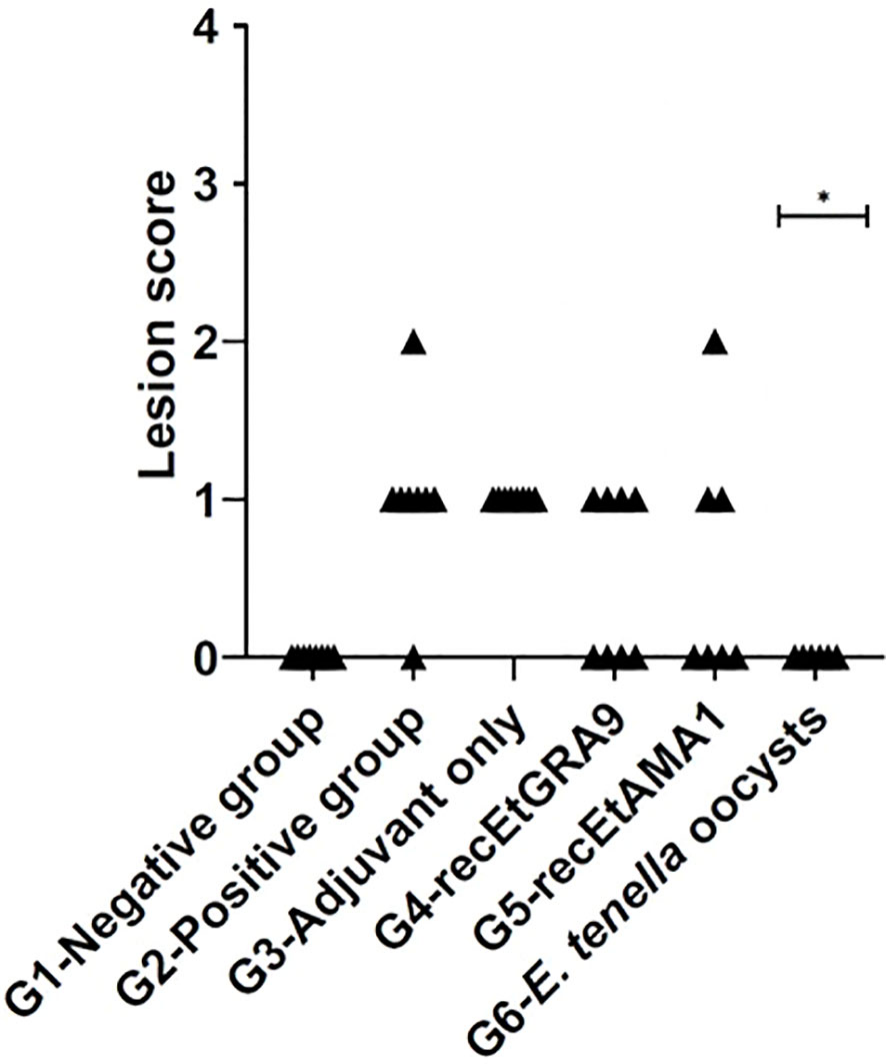
Caecal lesion scores five days after the challenge. Experimental groups are shown on the x-axis, and lesion scores are shown on the y-axis. Each marker represents one chicken (n=6-8). (*) Groups with significantly different mean lesion scores compared with the equivalent unvaccinated, challenged chicken group (G2) (positive control).

No significant difference in caecal lesion severity was detected between the unvaccinated challenged control group and any of the recombinant protein vaccinated groups, although higher proportions of individuals presented with scores of 0 in the EtGRA9 and EtAMA1 vaccinated groups (G2 compared to G3-5, **Figure 6**). Contrastingly, no caecal lesions were detected in chickens vaccinated by prior *E. tenella* oocyst exposure following challenge (G6, **Figure 6**).

### 3.9 *E. tenella* replication was reduced by vaccination but varied between caeca in individual chickens

**Figure 7:**
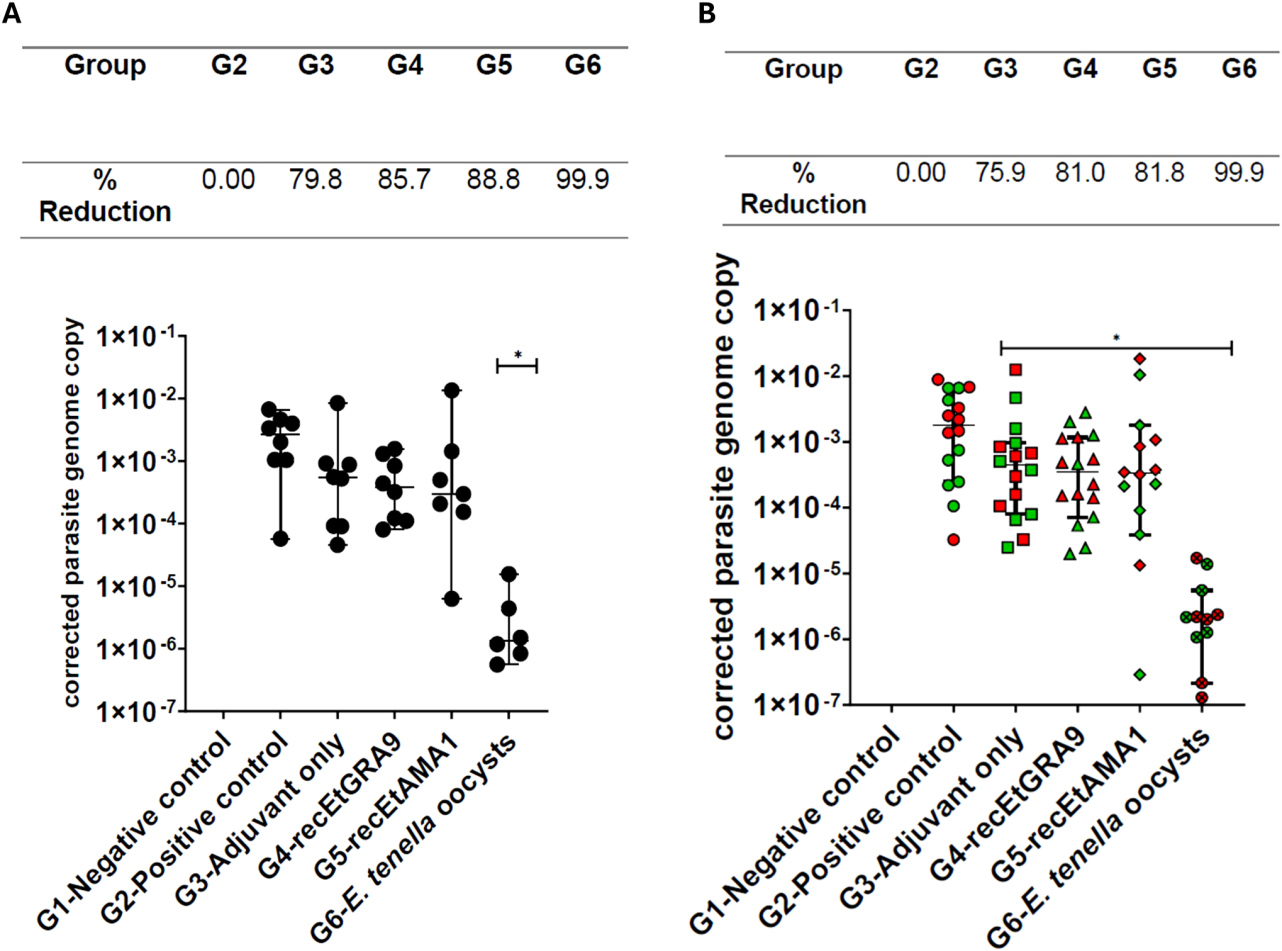
Parasite genome copy number assessed by qPCR of genomic DNA extracted from caeca following challenge, corrected using chicken TBP copy number for each chicken. Each marker represents an individual chicken (**A**: n=6–8 per group) or caeca (**B**: left: green; right: red; n=12–16 per group). Experimental groups are shown on the x-axis, and the corrected *E. tenella* genome copy number is shown on the y-axis. Each group’s median and 95% confidence interval (CI) are shown. Groups with significantly different median parasite genome copy numbers compared to the unvaccinated challenged chicken group (G2) are indicated with an asterisk (*). The percentage reduction in parasite load compared to the unvaccinated, challenged chicken group (G2) is shown above the graphs.

Caecal parasite load at five days post-infection was assessed by quantitative PCR, combining values from the left and right caeca from each individual as a single data point per chicken. A statistically significant decrease in parasite load (expressed as parasite genome copy number per host genome copy number) was only observed for the group vaccinated by prior *E. tenella* infection (G6) when compared to the unvaccinated positive control (G2) (99.9% reduction; **Figure 7A**). Parasite load was reduced in the adjuvant-only (79.8% reduction; G3), recEtGRA9 (85.7%; G4) or recEtAMA1 (88.8%; G5) immunised groups compared to G2, but high intra-group variation precluded statistical significance. When left and right caeca parasite replication was analysed separately, comparable average reductions in parasite replication were observed across groups, with reduced intra-group variation. Thus, parasite replication was significantly reduced in the adjuvant-only, recEtGRA9, recEtAMA1, and prior *E. tenella* infection-vaccinated groups (G3-G6) compared to the unvaccinated challenged control group (G2) (**Figure 7B**).

## 4 Discussion

Dense granule proteins (GRAs) secreted by apicomplexan parasites are essential for host cell invasion, with decisive roles in establishing the intracellular niche (71). Notable variations in GRA repertoires between apicomplexan genera and species may be tailored to specific host-parasite interactions and life cycle requirements. GRAs have been extensively studied in *T. gondii*, where a large number are abundantly expressed during the tachyzoite stage, associated with the acute phase of infection (72). In contrast, both the number of expressed GRA genes and their expression levels are considerably lower in *T. gondii* sporozoites and merozoites (intestinal stages in the definitive host, equivalent to *Eimeria* sporozoites and merozoites) (73). This difference is likely to stem from the distinct mechanisms of replication employed by tachyzoites and merozoites [endodyogeny vs. endopolygeny (74)] as well as the requirement for prolonged tachyzoite survival within the parasitophorous vacuole (PV) before developing into tissue cysts and establishing the chronic phase of infection. Additionally, GRAs in *Toxoplasma* play a crucial role in supporting the long-term persistence of cysts. This, along with the significantly broader host range of *T. gondii* tachyzoites (75), could explain why the GRA repertoire identified in *Eimeria* spp. that infects chickens is approximately one-third the size of the *T. gondii* GRA repertoire. This limited repertoire was further evidenced by the fact that only twelve GRAs have been annotated for *E. tenella* in the ToxoDB database, less than a sixth of the TgGRAs annotated in the *T. gondii* genome.

Comparative genetic analysis of EtGRA9, EtGRA12a and EtGRA12b with their orthologues in other *Eimeria* spp., *C. cayetanensis*, and *T. gondii*, revealed relationships consistent with previous studies at other loci (63, 76, 77). Amino acid sequences from *E. necatrix* and *E. tenella* were more closely comparable to each other than the other *Eimeria* species that infect chickens, supporting suggestions that at least two distinct ancestral *Eimeria* lineages may have given rise to the species infecting chickens today (77).

Analysis of EtGRA proteins revealed distinct structural features. Like other GRAs (NcGRA2, TgGRA9, NcGRA9, TgGRA12, TgGRA6, TgGRA7, TgGRA14), EtGRA9 is a small-sized protein (29 kDa). *etgra9* was found to have two introns, to be predominantly hydrophilic (although hydrophobic regions are also present) and to contain a putative N-terminal hydrophobic signal peptide of 23 amino acids. These findings are slightly different to what was first found by Adjogble et al. (78) when this group described GRA9 from *Toxoplasma*; or Leineweber et al. (50) to its orthologue in *Neospora caninum*, which contained only one intron. Beyond these differences, the overall characteristics remain consistent. TgGRA9 has been described as predominantly hydrophilic, although short hydrophobic α-helices have also been identified (78). Similarly, Leineweber et al. (50) characterised NcGRA9 as hydrophilic with shorter hydrophobic regions. In the case of EtGRA9, multiple α-helical regions were identified, three of which exhibited amphiphilic properties, suggesting a potential role in protein-lipid interactions. These structural features are commonly observed among secreted GRA proteins in *T. gondii* and *N. caninum* (79). In contrast, EtGRA12a is a slightly larger protein (46 kDa) with one intron, hydrophilic properties, and a predicted transmembrane domain (TM), although this prediction is weak. ToxoDB default analysis initially suggested the presence of a signal peptide for this protein, but the TM domain may overlap with the potential signal peptide. ToxoDB may not have accurately identified the N-terminus, possibly due to a frameshift or sequencing error, which could have impacted the determination of the true initiation codon, likely positioned upstream. To further clarify, SignalP 6.0 prediction was run, which confirmed that EtGRA12a does not have a signal peptide. EtGRA12b shares similar characteristics to EtGRA12a but lacks the putative TM domain. Similar to *Toxoplasma*, a comparable percentage of their amino acid segment was predicted to adopt an alpha-helical secondary structure.

Antigenicity plots suggest strong immunogenic potential for all selected EtGRAs, consistent with findings in *T. gondii*. Immunisation with recombinant TgGRA9 and TgGRA12 in mice has resulted in significant immune responses, marked by elevated levels of the pro-inflammatory cytokines interferon-gamma (IFN-γ) and interleukin-12 (IL-12) (80). Additionally, Majidiani et al. (81) developed a multi-epitope vaccine using MHC-binding and B-cell epitopes from TgGRA12, TgSAG1, TgROP16 and TgMIC4. This vaccine significantly increased specific antibody levels against these proteins, enhanced IgG2a production, and promoted a Th1 immune response characterised by elevated IFN-γ levels. While immunogenicity is important for vaccine development against apicomplexans, it is not the sole factor for stimulating protection. Nevertheless, our findings suggest that GRAs in *Eimeria* could serve as potential candidates for vaccine development.

The structural predictions for EtGRA9, EtGRA12a, and EtGRA12b, generated using AlphaFold and I-TASSER, offer complementary insights. AlphaFold, utilising advanced deep-learning algorithms, provides highly accurate atomic-level predictions, especially for proteins without experimental data (59). In contrast, I-TASSER relies more on template-based modelling, effectively identifying biologically significant features like binding sites and enzyme catalytic sites (EC), which are important for understanding protein function and interactions (82).

In chickens, variations in the MHC-B region, particularly the peptide-binding specificity of BF2 (MHC class I), are linked to resistance or susceptibility to pathogens (83–85) and vaccine responses (86, 87). However, the specific B-haplotypes influencing resistance to *Eimeria* are not fully understood and can vary with factors like age, immune competence and genetics (88, 89). From the four B-haplotypes available to predict binding to MHC class I molecules (www.syfpeithi.de), this study found that EtGRA9 contains epitopes predicted to bind B-F4, B-F12, and B-F19, but no B-F15. However, this does not rule out the presence of epitopes binding other haplotypes not available for prediction analysis. Research on VALO Specific Pathogen Free (SPF) chickens identified B-F15, B-F19, and B-F21 as the most frequent B-haplotypes (90). However, dominant B-haplotypes in commercial layers (as Hy-Line hybrids used in this trial), likely to present mixed B-haplotypes due to crossbreeding for production traits, remain largely uncharacterised. Hence, the relevance of these predicted epitopes would depend on the main B-haplotypes circulating in commercial flocks.

Antibodies are thought to play a minor role in the protective immune response against *Eimeria* spp. infections in chickens (91), with T-cell-mediated immune responses found to be more important (92–95). Nonetheless, the recognition of recEtGRA9, recEtGRA12a and recEtGRA12b expressed as recombinant proteins by sera from chickens previously infected with *E. tenella* oocysts evidenced that this antigen was naturally recognised by the immune system of chickens, leading to the activation of adaptive immunity mechanisms. The stronger recognition of recEtGRA9 compared to the three fragments suggests the presence of multiple linear epitopes that may act synergistically to elicit an immune response, rather than depending solely on conformational epitopes. Among the fragments, Fragment 2 was the most strongly recognised, despite having fewer predicted antigenic determinants than Fragment 3, which had the highest number. This indicates that a higher number of predicted antigenic sites does not necessarily correlate with increased recognition by sera from chickens previously infected with *E. tenella*. Additionally, this could reflect a more arbitrary measure of the level and duration of protein exposure to the host immune system, which may further justify the use of recEtGRA9 for the subsequent vaccination trial.

When selecting vaccine candidates, it is essential that they exhibit low genetic variability in field strains to reduce the potential for immune escape mechanisms. For this, *gra9*, *gra12a* and *gra12b* sequences from *E. maxima* and *E. tenella* were included in a SNP analysis. *E. tenella* showed lower diversity in *etgra* sequences, making them appealing vaccine candidates due to their potential immune recognition across strains and lower potential for vaccine escape. While no PCR errors were evident, *emgras* could also be promising candidates for *E. maxima*, although more SNPs were found for this species, already known for exhibiting a greater genetic and antigenic diversity (96, 97).

The immunisation of chickens with recEtGRA9 reduced parasite load five days post-challenge by 85.7% compared to unimmunised controls. This level of reduction closely mirrored that observed in chickens vaccinated with recEtAMA1 of 88.8%. Notably, this percentage exceeded reductions previously reported in similar studies, with values of 66% (31) and 64.7% (18), although the method of protein expression and delivery used differed in the latter experiment (*S. cerevisiae*). However, these results should be taken with caution, as a significant decrease in parasite load was also observed in the group that received only adjuvant for immunisation (79.8%). This showed a contribution of the adjuvant to the enhanced levels of protection achieved when immunising with each of the recombinant proteins. Adjuvants that activate pattern-recognition receptors (e.g., TLRs, NOD-like receptors) can stimulate innate immune responses and enhance resistance independently of antigen specificity (98). For example, TiterMax has been shown to activate T cells and induce cytokine-producing cells (e.g., IFN-γ and IL-4), supporting its role in promoting both cellular and humoral responses (99). Such activation may induce ‘trained immunity,’ a heightened innate state that reduces parasite replication even in the absence of specific antigens (100). Chickens vaccinated with live oocysts showed strong protection (99.9%), consistent with findings in other studies (18, 101). It is worth noting that, among the EtGRA-based vaccines for *Eimeria*, only recEtGRA12a has been studied and proven effective before, with a 29% reduction in parasite replication. This vaccine was also expressed as a recombinant protein in *E. coli* and administered intramuscularly (102).

This study used a dose for the challenge of 300 oocysts. This model minimizes the crowding effect and allows assessment of vaccine efficacy by linear quantification of parasite replication in caecal tissue, but induces limited pathology (103). Typically, higher challenge doses (>5,000 oocysts) are used to evaluate intestinal lesions and weight changes over a longer period (31, 103), but do not allow a linear quantification of parasite replication. Studies using various antigens and a high-dose challenge showed different levels of efficacy based on the percentage reduction in oocyst output: recEtSAG6 (63%), EtSAG16 (59%) (33), recEtGAM56 (52%) (104), recEtMIC2 (38%), recEtMIC7 (75%), recEtM2AP (88%) and recROPK-Eten5-A (82%) (33, 102). Alternative expression systems such as DNA vaccines or yeast have not significantly improved the reduction in oocyst output compared to the immunisation with a recombinant protein form. However, developing oral vaccines would enhance practicality in terms of easier administration and scalability for mass production, making them more suitable for widespread commercial use. Despite using a low dose, this study showed significant differences in body weight and lesion scores, with recEtGRA9-vaccinated chickens showing significantly improved weight gain compared to controls. Further research with higher challenge doses, larger experimental groups and alternative expression systems would allow assessment of its full potential to be developed as a recombinant commercial vaccine against chicken coccidiosis.

This study independently analysed *Eimeria* infection in both caeca and, although differences in parasite load between the left and right caecum were not significant, there was a trend of higher parasite loads in the right caecum (62.2%) vs. the left caeca (37.8%) (**Supplementary Tables 7, 8**). This could be due to biological asymmetry (105), variations in immune response (106), or differences in microenvironmental factors like pH (107), nutrient availability (108), or microbiota composition (109).

In summary, while controlling chicken coccidiosis remains challenging, this research paves the way for future studies in vaccine antigens, highlighting the need for further research into EtGRAs to uncover potential protein functions, drug targets, vaccine candidates and/or diagnostic markers (110, 111). Additionally, the study demonstrates that recEtGRA9 shows significant promise as a vaccine candidate as it reduced parasite loads and provided immunoprotection comparable to recEtAMA1, a widely used candidate in *Eimeria* vaccine development research to date (30, 31).

## Data Availability

The raw data supporting the conclusions of this article will be made available by the authors, without undue reservation.
