# Supplementary material for: Study of dense granule proteins in Eimeria spp. identifies a limited repertoire with potential as vaccine candidates
Source: Front Immunol. 2025 Jul 16;16:1605984. doi: 10.3389/fimmu.2025.1605984 (PMC12309413; doi:10.3389/fimmu.2025.1605984)
Supplement: Supplementary file 1 [file DataSheet1.docx]

Table I- Primers and conditions used for polymerase chain reaction (PCR): **A.** primers for full-length coding sequence amplification, cloning into pGEM T-easy and sequence analysis; **B.** primers and PCR conditions for amplification of etgra loci; **C.** primers for cloning into pET-32b (+) for recombinant protein expression in E. coli; **D.** primers for amplifying etgra9 fragments for pET-32b (+) constructs used in protein expression; and **E.** primers for parasite quantification by quantitative PCR (qPCR) in the vaccination trial.

**A. Primers for full-length coding sequence amplification, cloning into pGEM T-easy (Promega) and analysis of sequences.**

| *RE | Target locus | Primer sequence (5’-3’) | | Amplicon size (*bp) | Transformed to | Aim |
| --- | --- | --- | --- | --- | --- | --- |
| - | *etgra9* | gDNA | Fw: TGTTCTTTTGCCCGTCAG  Rv: GTCTGCACCTCTCCTCAA | 1035 | *Escherichia coli JM109* | *SNP |
|  | *etgra12a* |  | Fw: AGTCTTTAGTAATCCAGTGTTA  Rv: CCATACAGCTACATTTCG | 1432 |  |  |
|  | *etgra12b* |  | Fw: TTGTGGTGTGGGACCTTC  Rv: AGTGTAAATGCTCGATGG | 1337 |  |  |
| **XbaI | *etgra9* | cDNA | Fw: tctagaGTGCTCCATTGCGAGTGCAG  Rv: GCtctagaGTTCAGCACATCCA | 842 | *Escherichia coli JM109* | SNP |
|  | *etgra12a* |  | Fw: tctagaGTCGATCCTCGCAGCGT  Rv: GCtctagaATTTCTCTTCG | 1255 |  |  |
|  | *etgra12b* |  | Fw: tctagaGTGGATTGACTGGCTTTT  Rv: GCtctagaTGAGTCTCCC | 1221 |  |  |

| *Eimeria* spp. | Target locus | Primer sequence (5’-3’) (gDNA) | Field sample |
| --- | --- | --- | --- |
| *E. tenella*  **B. PCR material and conditions for amplification of subject *etgra* loci.** | *etgra9* | Fw: GTTCTTTTGCCCGTCAG  Rv: GTCTGCACCTCTCCTCAA | 22.2  22.4  23.2  23.3  25.2  26.1  26.3  27.1  27.2  137.7 |
|  | *etgra12a* | Fw: CTTTAGTAATCCAGTGTT  Rv: CCATACAGCTACATTTCG  Fw: GGGTGAAACGTGCTTTAG  Rv: TGTTCGGCTTCATCTCCC |  |
|  | *etgra12b* | Fw: TTGTGGTGTGGGACCTTC  Rv: AGTGTAAATGCTCGATGG  Fw: GGCGTCCTTGTTTTCTGT  Rv: GTTTGACACAGCACCGAG |  |
| *E. maxima* | *emgra9* | Fw: GCAGTTGCTACGAAAAAC  Rv: CCCCTGCGATTATTCTTG | 22.2  23.2  26.1  27.1  27.2  136.1  136.3  136.5  136.8 |
|  | *emgra12a* | Fw:TTTCTGCGATGCTTTCTC  Rv: CAGTATCATCCACTAAGG |  |
|  | *emgra12b* | Fw:GAAGTGCTGGATACTCAA  Rv: GATACAGCAAATACCGGG |  |

**C. List of primers used for amplification and cloning into pET-32b (+) for recombinant expression.**

| Primer name | Target locus | Primer sequence (5’-3’) | RE | Size (bp) | Cloning protocol |
| --- | --- | --- | --- | --- | --- |
| Fw_G9_BamHI | *etgra9* | agaggatccaTGCGATTCAAGC | BamHI | 763 | Restriction enzyme homologous ends |
| Rv_G9_NotI |  | agcggccgcGTTCAGCACAT | NotI |  |  |
| G12A_fwd | *etgra12a* | acaaggccatggcgatatcgGATGTTGGCGTTTACTCTC | - | 1186 | Gibson assembly (seamless cloning) |
| G12A_rev |  | tggtggtggtgctcgagtgcATTTCTCTTCGAGGCCGAATTTC | - |  |  |
| G12B_fwd | *etgra12b* | acaaggccatggcgatatcgGACGTTGGTATATTTTCTTCC | - | 1153 |  |
| G12B_rev |  | tggtggtggtgctcgAGTGCTGAGTCTCCCTTACTCCTC | - |  |  |

| Peptide | Position (bp) | Length (bp) | Length (*aa) | Molecular weight  (Da) | Primer sequence (5’-3’) | RE | Epitope number |
| --- | --- | --- | --- | --- | --- | --- | --- |
| 1^st^ Fragment | 70-268 | 198 | 66 | 6,936 | Fw:AGAggatccATGCGATTCAAGC  Rv:gcggccgcTGCGAGAGAAA | BamHI  NotI | 1 |
| 2^nd^ Fragment  **D. Primers used to amplify etgra9 fragments to be cloned into pET-32b (+) constructs for protein expression.** | 256-486 | 231 | 77 | 8,680 | Fw:CGggatccAGTTTCTCTCGCA  Rv:TgcggccgcCGCAATGCAC | BamHI  NotI | 4 |
| 3^rd^ Fragment | 490-813 | 324 | 108 | 11,353 | Fw:CGAggatccATCTCCCGGG  Rv:AgcggccgcGTTCAGCACAT | BamHI  NotI | 1 |

| Primer name | Target locus | Primer sequence (5’-3’) |  | Aim | Reference |
| --- | --- | --- | --- | --- | --- |
| Fw_RAPD | *E. tenella* RAPD-SCAR marker Tn-E03-116 | TCGTCTTTGGCTGGCTATTC |  | Parasite DNA quantitation | (Blake et al., 2008) |
| Rv_RAPD |  | CAGAGAGTCGCCGTCACAGT |  |  |  |
| Fw_TBP | Chicken tata-binding protein | TAGCCCGATGATGCCGTAT |  | Host cell DNA quantitation | (Nolan et al., 2015) |
| Rv_TBP |  | GTTCCCTGTGTCGCTTGC |  |  |  |

**E. Primers used for parasite quantification by quantitative PCR (qPCR) in the vaccination trial.**

**BP: Base pairs; SNP: Single Nucleotide Polymorphism;RE: Restriction enzyme; AA: amino acids. ** Included in primers for cloning in alternative vectors. Capital letters: sequence; lowercase letters: restriction sites or overlapping annealing sites. PCR conditions: Initial denaturation: 1 × (94°C, 2 min); Denaturation & Annealing: 30 × (94°C, 30 s; Table 1A & 1C: 54°C, Table 1B: 52°C, Table 1D: 58°C, Table 1E: 56°C); Extension: 1 × (72°C, 10 min).*

Table II- Details of the field samples used for E. tenella and E. maxima loci amplification. Samples described previously in Clark et al. (2016).

| Sample ID | Country of origin | Number of oocysts in volume of sample used for DNA extraction | *Eimeria* spp. represented |
| --- | --- | --- | --- |
| 22.1 | Denmark | 1,000,000 | *E. tenella, E. acervulina* |
| 22.2 | Denmark | 1,000,000 | *E. tenella, E. acervulina, E. maxima* |
| 22.4 | Denmark | 1,000,000 | *E. tenella* |
| 23.2 | Poland | 1,000,000 | *E. tenella, E. acervulina, E. maxima* |
| 23.3 | Poland | 1,000,000 | *E. tenella* |
| 25.2 | New Zealand | 1,000,000 | *E. tenella, E. acervulina* |
| 26.1 | Poland | 1,000,000 | *E. tenella, E. acervulina, E. maxima* |
| 26.3 | Poland | 314,000 | *E. tenella, E. acervulina* |
| 27.1 | Italy | 1,000,000 | *E. tenella, E. maxima* |
| 27.2 | Italy | 1,000,000 | *E. tenella, E. acervulina, E. maxima* |
| 136.1 | Portugal | 140,295 | *E. tenella, E. acervulina, E. maxima* |
| 136.2 | Denmark | 43,095 | *E. tenella* |
| 136.3 | Unknown | 119,340 | *E. tenella, E. acervulina, E. maxima* |
| 136.4 | Unknown | -* | *E. tenella, E. acervulina* |
| 136.5 | Unknown | - | *E. tenella, E. acervulina, E. maxima* |
| 136.6 | Italy | 46,410 | *E. tenella* |
| 136.7 | Philippines | 14,733 | *E. tenella, E. acervulina* |
| 136.8 | Netherlands | - | *E. tenella, E. acervulina, E. maxima* |

** -: sample contained an abundance of debris, which complicated oocyst counting.*

**A** (816 bp)

ATGGCTCCATTGCGAGTGCAGTTTGCTTCTGTTGCTGCTCTGATGGCAGCACACCTGTCCTTCGCCAGTTGCGATTCAAGCGAACGAAGGAGCATTGTGGAACCTGAGAAGCCAGAGCCTCTGGACATTGGCGGGTTCTTTGGTGGCGGGTCGTTTGCAGAGCTGGACAGGCTTTTCCAGCAGTCCATGTCGTCTCTCTTCGGTGTGCTGGGCCCAATGGGTGGTATGATGCAGCCAGTGGGGGCGGATCTGTCGGTTTCTCTCGCAGAGGGTGATGAGCGCACATGTCAATTCCGAGTCAAGATGGGTGATAACAGCGTCTCAATGAACAGCGTCACGTTGGGGGTGGACTACAACGGAAAATTCCTGTTGGCGAGTATCCACCGAGAACAGACGCGTAAGGAGCACGGGGAGAAGGGCGAGAGCCTCTTCTCCCGCAGCTTCCATGTGCGCAGTACGGTGCACCTGCCCGAAAGGTGCATTGCGACTCCCGGGGTGCTTCTGGCGAGTCTGGCGGGATATATGGTGAGCTCGAGCGGGTCGGAGGCCATGGTTGTGTTCCCATCCACCGTGCTGTTGAATGAGGGTGTCGAGAAGGGTTTGTTGCCAGAAAATATTGCAGAGAGTGTTACAAGGGGGGACCAACGCAGCATCAAGGATCTGACGTCAGCTCAGCAGTGCCTTGCTGCGGGGTTCACCGTGGAGCAGTGCAGCAAGTTGGGGGAGACCAAGCCACAGGTTTCCTTGGTGAAGCCTACAGACGGCGGATACGTTCCCGTTCCCCGTTTCGATGTTGAACTGGATGTGCTGAACTAA

**B** (271 aa)

MAPLRVQFASVAALMAAHLSFASCDSSERRSIVEPEKPEPLDIGGFFGGGSFAELDRLFQQSMSSLFGVLGPMGGMMQPVGADLSVSLAEGDERTCQFRVKMGDNSVSMNSVTLGVDYNGKFLLASIHREQTRKEHGEKGESLFSRSFHVRSTVHLPERCIATPGVLLASLAGYMVSSSGSEAMVVFPSTVLLNEGVEKGLLPENIAESVTRGDQRSIKDLTSAQQCLAAGFTVEQCSKLGETKPQVSLVKPTDGGYVPVPRFDVELDVLN

Figure I- EtGRA9 cDNA (**A**) and protein (**B**) sequences. Selected recEtGRA9 fragments are indicated by colours: recEtGRA9-F1 – grey; recEtGRA9-F2 – yellow; recEtGRA9-F3 - green. Underlined sequence indicates overlapping sequence between F1 and F2. Signal peptide region (purple) and stop codon (red) were excluded from cloning.

Table III- GRA proteins annotated in Toxoplasma gondii genomes and their homologues in Eimeria spp. genome assemblies. GRA proteins are represented in ascending number order. Gene IDs, final cellular localisation for each T. gondii GRA protein, and the degree of aminoacid identity (%) when compared to the E. tenella orthologue are represented. PVM: Parasitophorous vacuole membrane; PVL: Parasitophorous vacuole lumen; IVN: Intravacuolar network; CW: Cyst wall.

Accession numbers correspond to the ToxoDB database (<https://toxodb.org>). The following genome assembly versions were used: *Toxoplasma gondii* GT1 GCA_000149715.2 (ToxoDB release 49, Nov 5, 2020); *Eimeria falciformis* Bayer Haberkorn GCA_002271815.1 (ToxoDB release 60, Nov 9, 2022); *Eimeria brunetti* Houghton, *Eimeria acervulina* Houghton, *Eimeria maxima* Weybridge, *Eimeria mitis* Houghton, *Eimeria necatrix* Houghton, *Eimeria praecox* Houghton, *Eimeria tenella* strain Houghton GCA_000499725.1, GCA_000499425.1, GCA_000499605.1, GCA_000499745.2, GCA_000499385.1, GCA_000499445.1, GCA_000499545.1, respectively (ToxoDB release 49, Nov 5, 2020).

Table IV- EtGRA9, EtGRA12a, EtGRA12b predicted secondary structures and features. Red: Helix; Blue: Strand; Black: Coil; Yellow highlight: Signal Peptide; Underlined: Transmembrane domain.

| Protein | Protein sequence |
| --- | --- |
| EtGRA9 | MAPLRVQFASVAALMAAHLSFASCDSSERRSIVEPEKPEPLDIGGFFGGGSFAELDRLFQQSMSSLFGVLGPMGGMMQPVGADLSVSLAEGDERTCQFRVKMGDNSVSMNSVTLGVDYNGKFLLASIHREQTRKEHGEKGESLFSRSFHVRSTVHLPERCIATPGVLLASLAGYMVSSSGSEAMVVFPSTVLLNEGVEKGLLPENIAESVTRGDQRSIKDLTSAQQCLAAGFTVEQCSKLGETKPQVSLVKPTDGGYVPVPRFDVELDVLN |
| EtGRA12a | MRSSQRRRLALFGALMVALFELVVADVGVYSPEGSMVRVFNTLGSNMWYMSAEGCRVGLPGNLVVTPRAGSNAEGGELLDPMFTGPHLCSWLSMMEKAHHSMKKAWEKEHERQRSKVPRWNLLRRYFMWKQAFPELVFDVEIRYIDLWNKDRFGLPLPWATALFRYRCPDSKTSYGLFEHLCGAVFTQSPDSRVPSEVYLLIQPRQGFNRPLQVSASNWQFVSGALAGLGSLRKNRSDEKEEESVGLLGTLKSLYTRHEAIMTIPGKINDYWMYAGRCYFKWLLRLQWGFFNETFCERLHQQASSGVLGAVKAAAVDSVQLHVVLLSLFRHETPLMYGAIDEGVLGLNEVSDLLTMEIDTSPQNVPDFERSKSTAENVRTANMIFNGVSAFLAKKPASKGNSASKRN |
| EtGRA12b | MGLTGFFILLGIVYSSLIIEKAIADVGIFSSDGNSIRTHSTLHRQSWTMSPDGCRVGKAGNLVVTPLGGSAADGREILDTQGTGPQLCAWLSMMEKAHYEMSSAWESRHNRKRSQVSRWNILRRFLLWTRSFPELEFQVEVRYISLWNKDRFGHPMPWATAVFRYICPQSERSYGLLDHLCGAVFSQGKDPRVGTEVYLLLQPRTGYNRPLDVRASNWQYVSGALSGLRGARTESQREADEKWMWLSSTFSRLKSIMTITGKVDDCWMNGGECYFKWLLRVEWRLFSETVCERLHRDANAGFGGALKAAALESVQVNITGMDLFNFSSPLLYGVLDEGMLQLNAGSGFVALEVDTNPFNPPNFERAKSTSQNVRAASMLFKGVRSFFGARSKGDS |


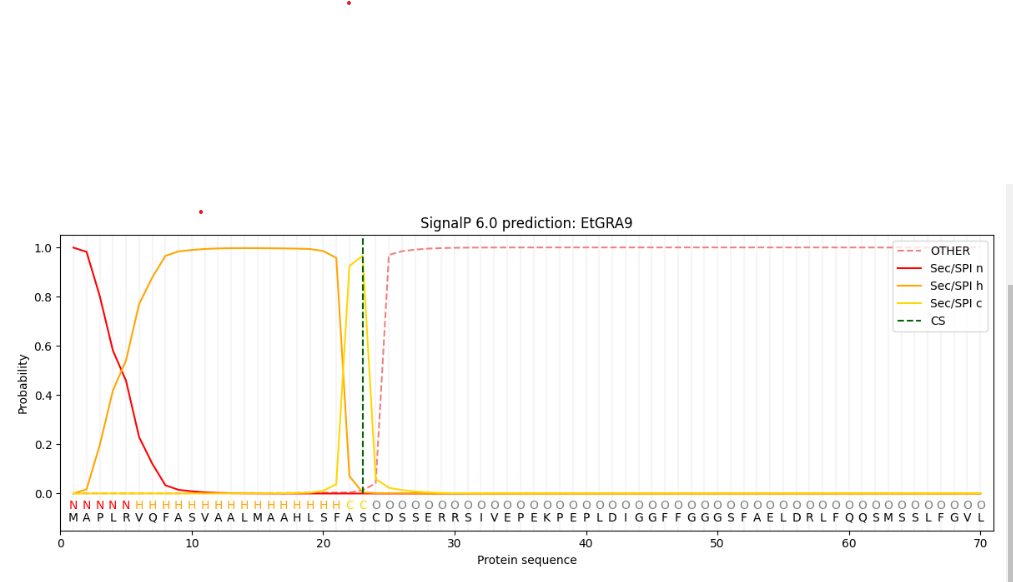

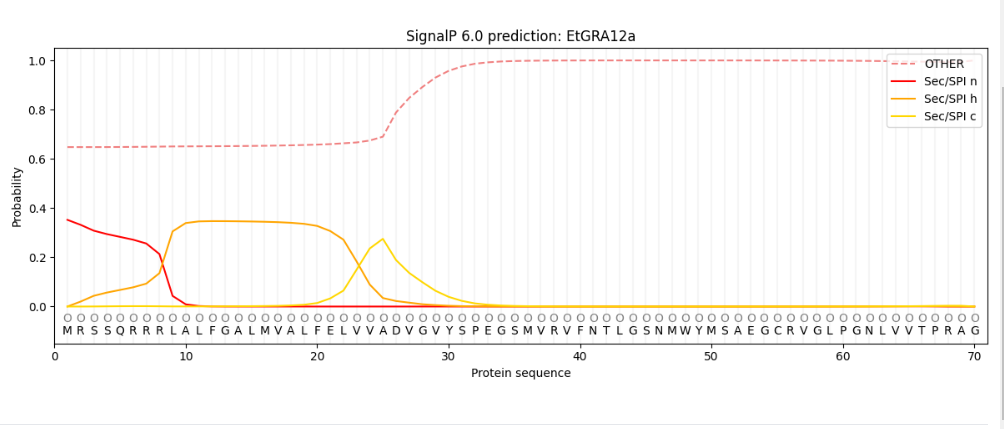


**B**

**D**

**A**

**D**


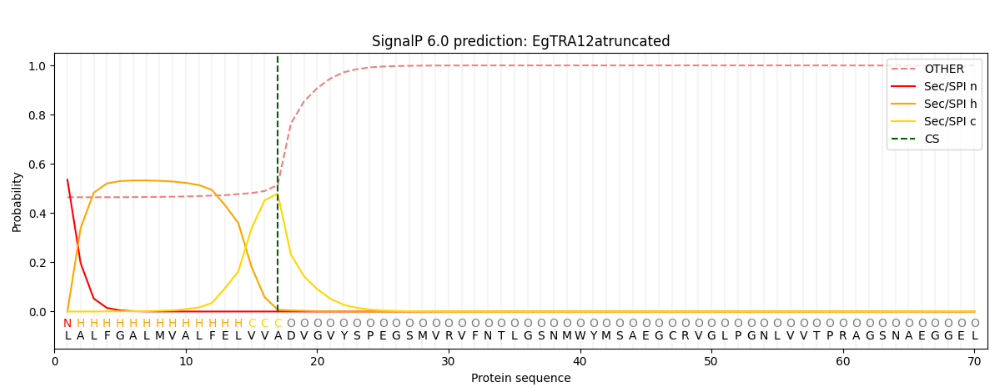


**D**

**D**

**C**

**D**


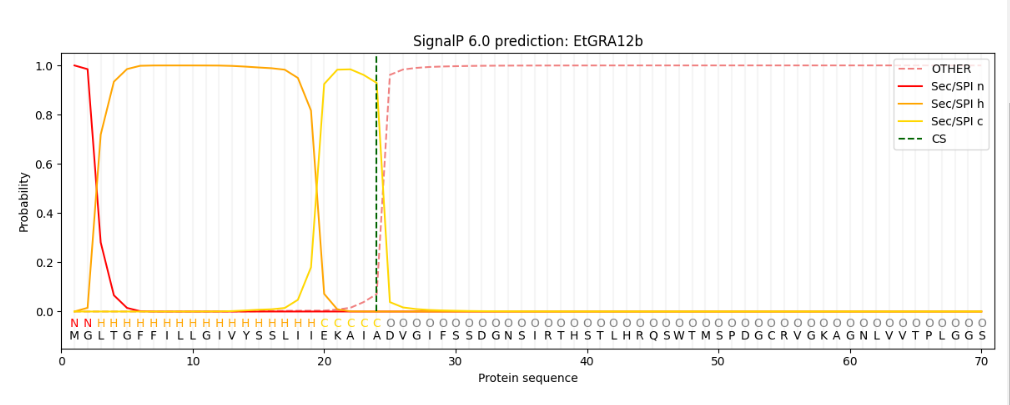


Figure II- Signal peptide prediction for EtGRA9 (**A**), EtGRA12a (**B**), truncated EtGRA12a (**C**) and EtGRA12b (**D**) with SignalP 6.0 software.


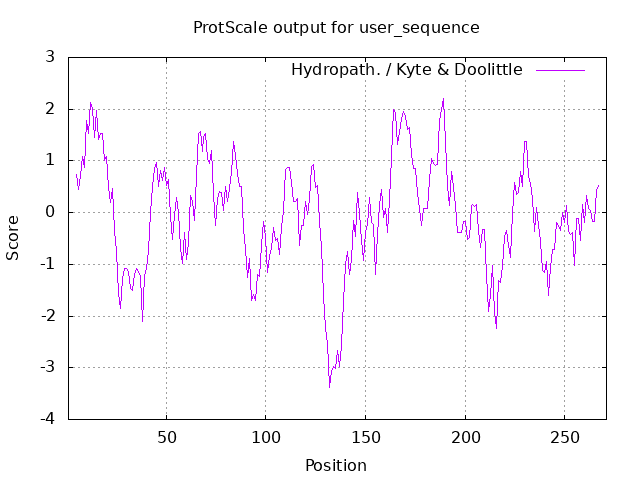

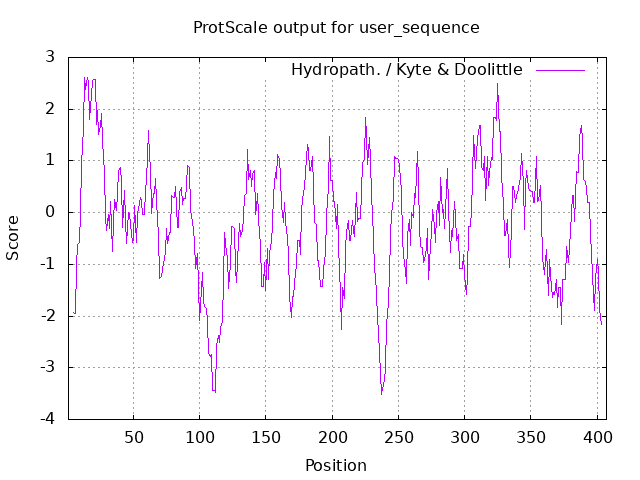

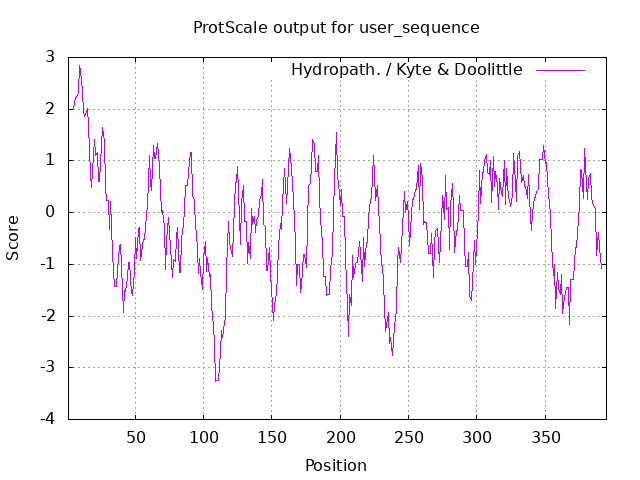


**A**

**D**

**B**

**D**

**C**

**D**

Figure III- Hydrophobicity plots for EtGRA9 (**A)**, EtGRA12a (**B)** and EtGRA12b (**C)**, generated by ProtScale using the algorithm developed by Kyte and Doolittle (1982). The amino acid position is represented in the “X” axis, and the hydrophobicity score is represented in the “Y” axis (>0 = hydrophobicity; <0=hydrophilicity). A balanced distribution of hydrophobic and non-hydrophobic regions was observed for all three proteins resulting in an overall neutral profile.


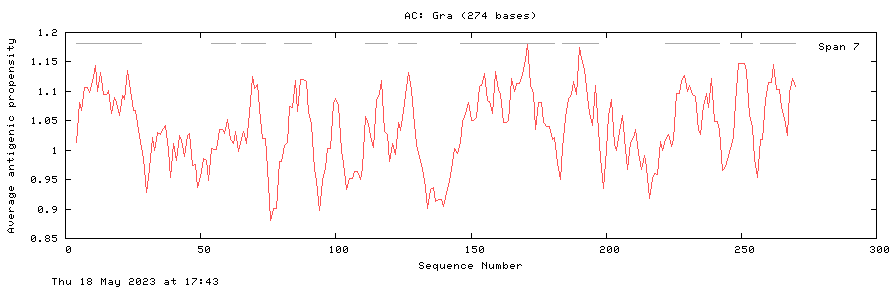

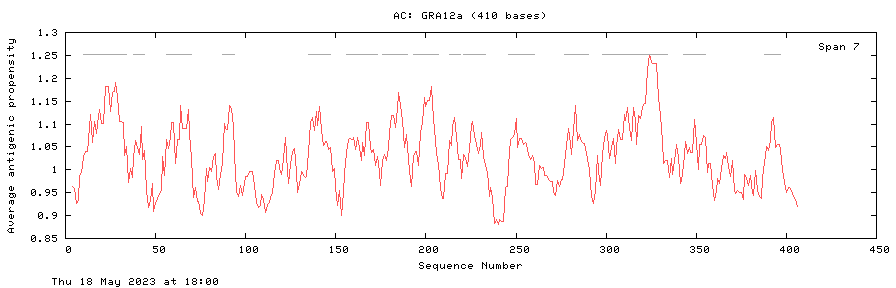


**C**

**D**

**B**

**D**

**A**

**D**


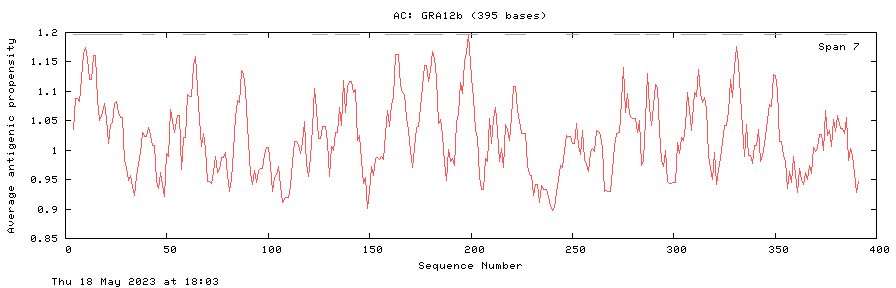

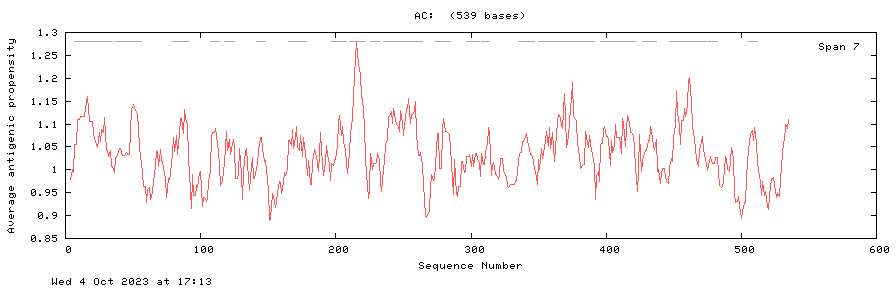


**D**

**D**

Figure IV- Antigenicity plot for EtGRA9 (**A)**, EtGRA12a (**B**), EtGRA12b (**C**) and EtAMA1 (**D**) generated using the using the method of Kolaskar and Tongaonkar (1990). Sequence amino acid number is represented in the X axis, whereas average antigenic propensity is represented in the Y axis.

Table V- Antigenic determinants for EtGRA9, EtGRA12a and EtGRA12b identified using the method of Kolaskar and Tongaonkar (1990). Starting and ending amino acid positions and sequences are indicated.

|  | n | Start Position | Sequence | End Position |
| --- | --- | --- | --- | --- |
| EtGRA9 | 1 | 4 | MAPLRVQFASVAALMAAHLSFASCD | 28 |
|  | 2 | 54 | SFAELDRLFQ | 63 |
|  | 3 | 65 | SMSSLFGVLG | 74 |
|  | 4 | 81 | QPVGADLSVSL | 91 |
|  | 5 | 111 | SMNSVTLGV | 119 |
|  | 6 | 123 | GKFLLASI | 130 |
|  | 7 | 146 | LFSRSFHVRSTVHLPERCIATPGVLLASLAGYMVSS | 181 |
|  | 8 | 184 | SEAMVVFPSTVLLN | 197 |
|  | 9 | 222 | KDLTSAQQCLAAGFTVEQCSK | 242 |
|  | 10 | 246 | TKPQVSLVK | 254 |
|  | 11 | 257 | DGGYVPVPRFDVEL | 270 |
| EtGRA12a | 1 | 10 | RRLALFGALMVALFELVVADVGVYS | 34 |
|  | 2 | 38 | SMVRVFN | 44 |
|  | 3 | 56 | EGCRVGLPGNLVVTP | 70 |
|  | 4 | 87 | TGPHLCSW | 94 |
|  | 5 | 135 | AFPELVFDVEIRY | 147 |
|  | 6 | 156 | FGLPLPWATALFRYRCPD | 173 |
|  | 7 | 176 | TSYGLFEHLCGAVFT | 190 |
|  | 8 | 193 | PDSRVPSEVYLLIQP | 207 |
|  | 9 | 213 | RPLQVSA | 219 |
|  | 10 | 221 | NWQFVSGALAGLG | 233 |
|  | 11 | 246 | ESVGLLGTLKSLYTR | 260 |
|  | 12 | 277 | YAGRCYFKWLLRLQ | 290 |
|  | 13 | 298 | FCERLHQQASSGVLGAVKAAAVDSVQLHVVLLSLFRH | 334 |
|  | 14 | 343 | IDEGVLGLNEVSD | 355 |
|  | 15 | 388 | FNGVSAFLAK | 397 |
| EtGRA12b | 1 | 4 | TGFFILLGIVYSSLIIEKAIADVGI | 28 |
|  | 2 | 38 | THSTLHR | 44 |
|  | 3 | 58 | KAGNLVVTPLGG | 69 |
|  | 4 | 83 | TGPQLCAW | 90 |
|  | 5 | 122 | LRRFLLWT | 129 |
|  | 6 | 133 | PELEFQVEVRYIS | 145 |
|  | 7 | 158 | WATAVFRYICPQ | 169 |
|  | 8 | 172 | RSYGLLDHLCGAVFS | 186 |
|  | 9 | 193 | VGTEVYLLLQP | 203 |
|  | 10 | 217 | NWQYVSGALSG | 227 |
|  | 11 | 247 | SSTFSRL | 253 |
|  | 12 | 271 | GECYFKWLLRVEW | 283 |
|  | 13 | 286 | FSETVCER | 293 |
|  | 14 | 304 | GALKAAALESVQV | 316 |
|  | 15 | 324 | FNFSSPLLYGV | 334 |
|  | 16 | 345 | GSGFVALEV | 353 |
|  | 17 | 375 | AASMLFKGVRS | 385 |

**A**

**D**


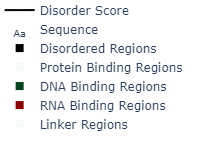

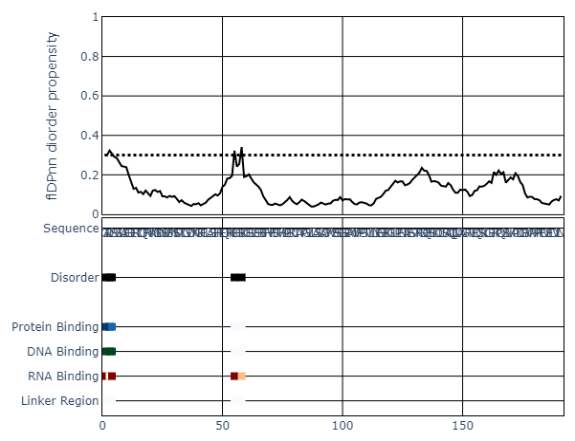

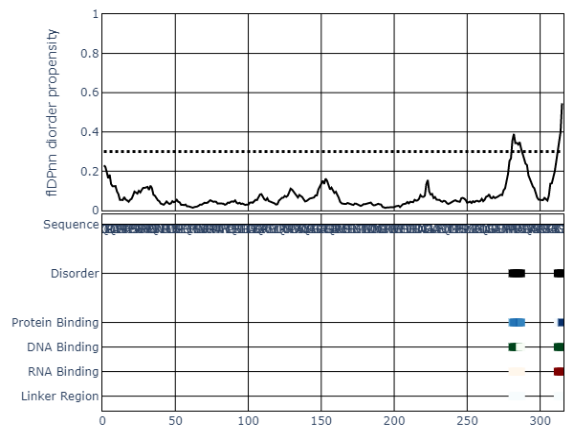

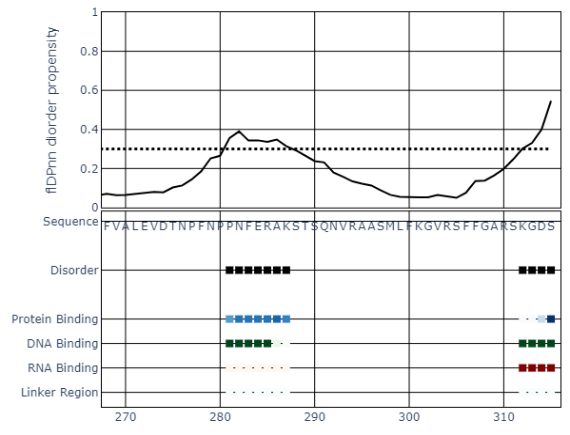

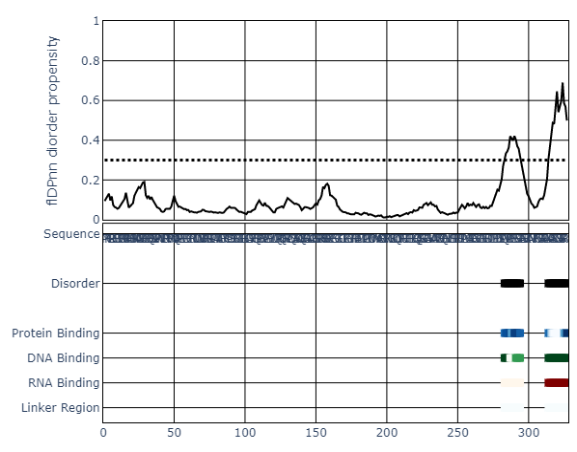

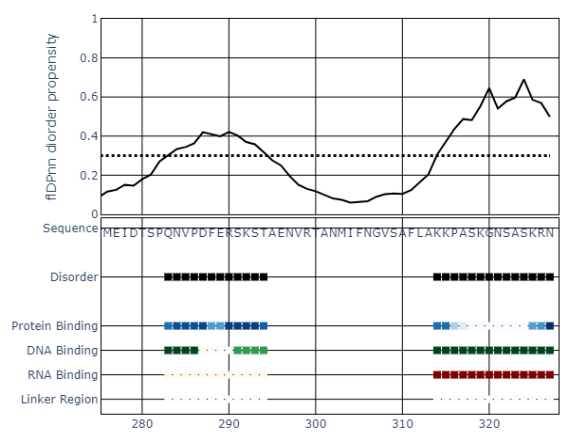


**B2**

**D**

**B1**

**D**

**C2**

**D**

**C1**

**D**

Figure V- Disordered region plot in **A.** EtGRA9, **B1.** and **B2**. EtGRA12a, and **C1**. and **C2.** EtGRA12b. The X axis represents the amino acid chain, whereas the Y axis shows the disorder propensity (flDPnn). The plots on the right (**B2** and **C2**) provide a magnified view of the disordered regions circled in red in graphs **B1** and **C1** for EtGRA12a and EtGRA12b, respectively.

**A**

**D**


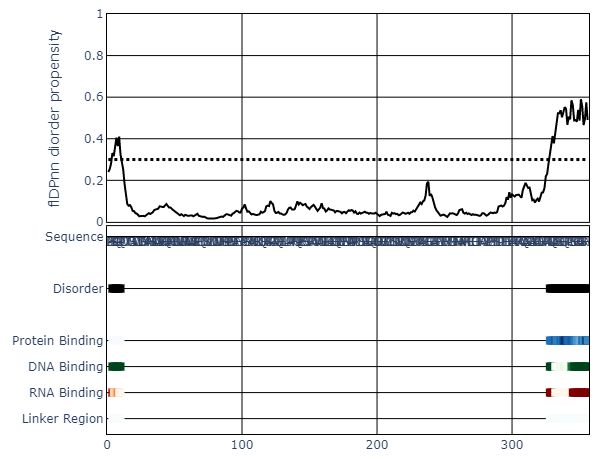

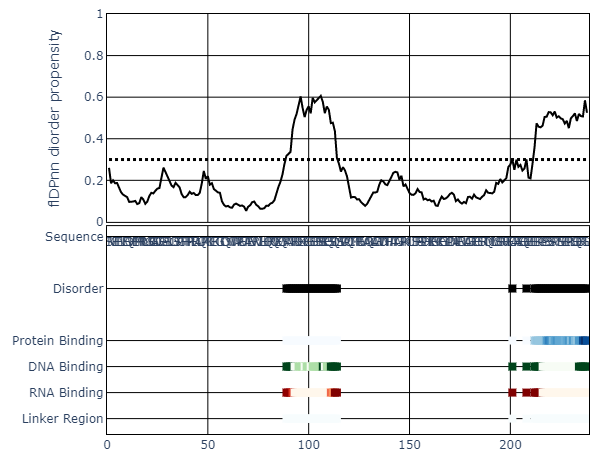

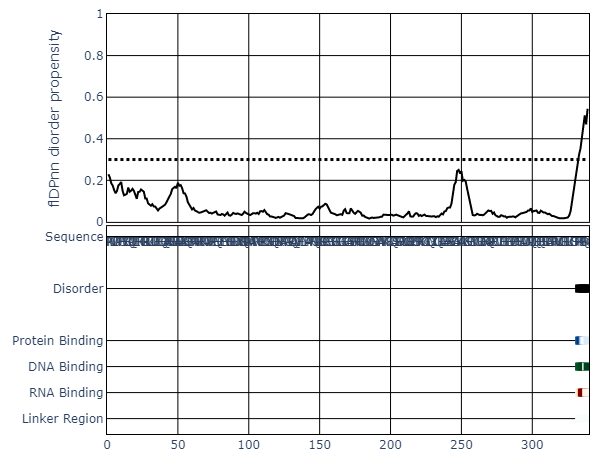


**B**

**D**

**C**

**D**

Figure VI- Disordered regions plot in **A.** TgGRA9, **B.** TgGRA12a and **C.** TgGRA12b. The X axis represents the amino acid chain, whereas the Y axis shows the disorder propensity (flDPnn).

**E**

**D**

**C**

**D**

**G**

**F**

**F**

**F**

**E**

**F**

**D**

**D**

**C**

**C**

**B**

**B**

**A**

**A**


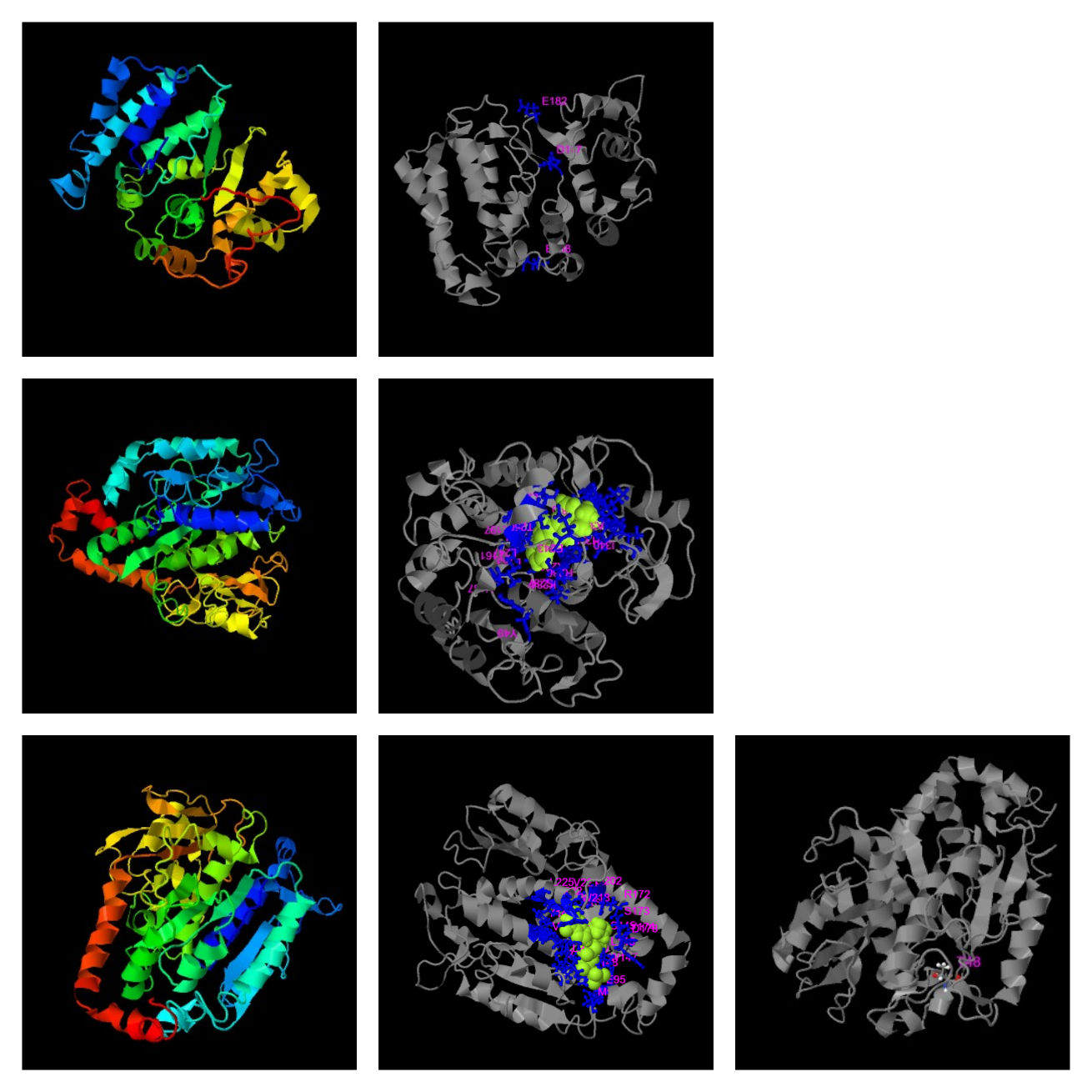


**B**

**D**

**C**

**D**

**D**

**D**

**A**

**D**

Figure VII- Predicted ligand binding sites for EtGRA9 (**A**), EtGRA12a (**B**) and EtGRA12b (**C**) using I-TASSER server. Enzyme Commission (EC) numbers and active sites for EtGRA12b (**D**) using I-TASSER server. Enzyme name: Inositol-3-phosphate synthase. Reaction catalyzed: D-glucose 6-phosphate <=> 1D-myo-inositol 3-phosphate.


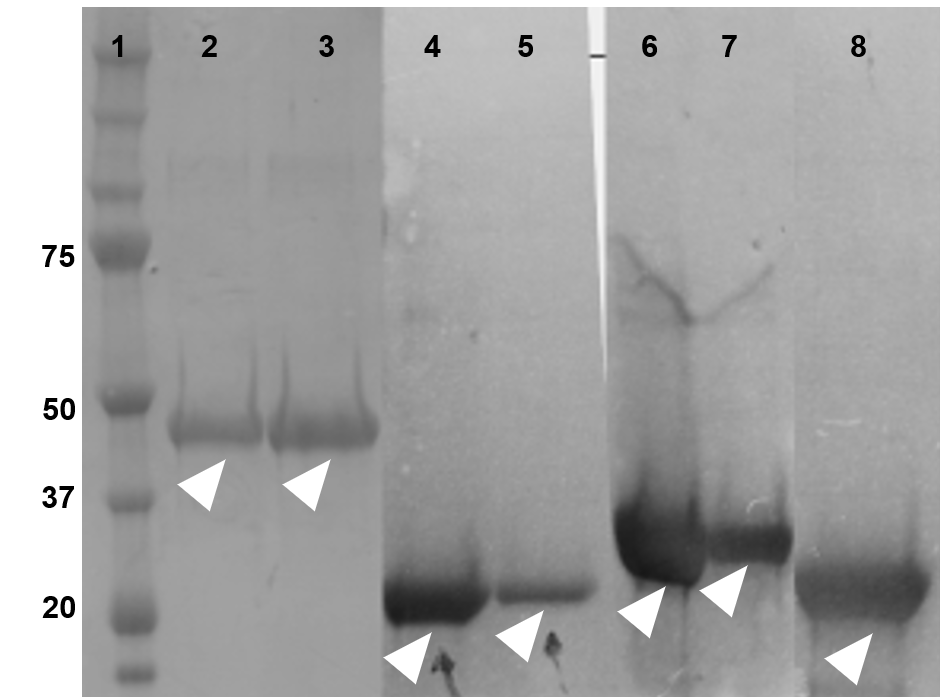

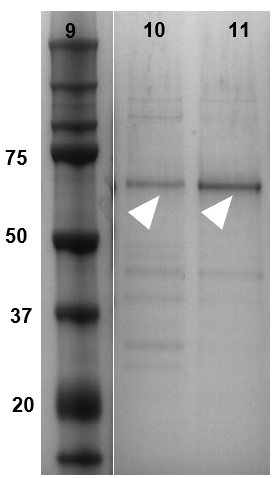

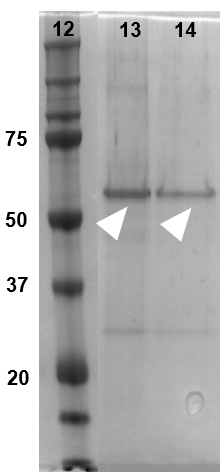

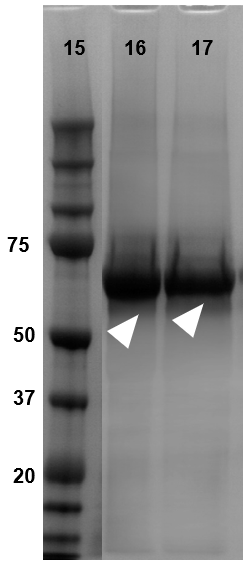


Figure VIII- Coomassie-stained gel showing the purification of EtGRA9 full length and fractions (lanes 1-8), EtGRA12a (lanes 9-11), EtGRA12b (lanes 12-14, and EtAMA1 (lanes 15-17). Lanes 1, 9, 12 and 15 represent molecular weight markers, with molecular weights indicated in kDa on the left side of the gel. Proteins of interest (indicated by white arrows) were detected in the elution fractions.

Table VI- Summary of chicken body weight during the vaccination study. Chickens were weighed at day 7 (D7) (pre-immunisation), day 14 (D14), day 21 (D21), day 35 (D35) (before challenge) and day 40 (D40) (5 days after challenge).

| Group | Mean Body Weight (g, SD) | | | | | Mean Body Weight Gain (g, SD) | | *Weight Gain (%) |
| --- | --- | --- | --- | --- | --- | --- | --- | --- |
|  | **D7** | **D14** | **D21** | **D35** | **D40** | **Pre-challenge (D7-D35)** | **Post-challenge (D35-D40)** | **Post-challenge (D35-D40)** |
| G1-Negative control | 52.3, (2.1) | 96.7, (7.4) | ^a^188.1, (10.2) | 431.1(27.1) | 593.3, (29.6) | 378.7, (24.9) | ^a^162.2, (2.5) | 48.5 |
| G2-Positive control | 54.7, (3.8) | 94.7, (7.1) | ^a^191, (11.1) | 424, (14.2) | 533.2, (20.1) | 369.2, (10.3) | ^b^109.2, (5.9) | 0 |
| G3- Adjuvant only | 53, (1.7) | 91, (6.6) | ^a^182, (18) | 410.5, (40.4) | 527.7, (58.2) | 357.5, (38.7) | ^b^117.2, (17.7) | 7.3 |
| G4-recEtGRA9 | 55, (4.4) | 89.25, (9.7) | ^a^186.2, (17.3) | 430.3, (36) | 570.5, (44.8) | 375.3, (31.5) | ^a,c^140.1, (8.8) | 28.3 |
| G5-recEtAMA1 | 53, (4) | 97.1, (7.5) | ^a^188.5, (18.1) | 439.7, (32) | 564.2, (41.2) | 386.7, (27.9) | ^b,c^124.5, (9.2) | 14 |
| G6-*E. tenella* oocysts | 55.1, (3) | 95, (11.3) | ^b^158.6, (14.8) | 403.3, (28.9) | 517.5, (35.7) | 348.1, (25.9) | ^b,d^114.1, (6.7) | 4.4 |
| F statistic, p Value (ANOVA) | 1.082, 0.38 | 1.027, 0.41 | **3.404, 0.01** | 1.169, 0.34 | **3.430, 0.01** | 1.330, 0.27 | **14.11, <0.0001** | **-** |

**Weight gain percentage has been calculated compared to the challenged control group (G2).* ***Bold*** *indicates a significant difference between groups; groups marked with different letters were significantly different (P < 0.05).*

Table VII- Summary of comparative parasite presence in left and right caeca.

| **Group** | **Bird**  **number** | **Left/Right ratio*** | **Predominant**  **parasite presence** | **Interatio SD** | **High ratio (>3)** |
| --- | --- | --- | --- | --- | --- |
| **G2**  **(positive**  **control)** | 9 | 0.97 | Right caeca | 0.03 | no |
|  | 10 | 0.11 | Right caeca | 4.37 | **yes** |
|  | 11 | 0.54 | Right caeca | 0.65 | no |
|  | 12 | 0.16 | Right caeca | 3.02 | **yes** |
|  | 13 | 3.26 | Left caeca | 1.48 | no |
|  | 14 | 4.48 | Left caeca | 2.13 | no |
|  | 15 | 1.72 | Left caeca | 0.57 | no |
|  | 16 | 0.02 | Right caeca | 20.32 | **yes** |
| **G3**  **(adjuvant only)** | 17 | 0.75 | Right caeca | 0.29 | no |
|  | 18 | 1.14 | Left caeca | 0.13 | no |
|  | 19 | 0.16 | Right caeca | 3.12 | **yes** |
|  | 20 | 0.37 | Right caeca | 1.15 | no |
|  | 21 | 2.01 | Left caeca | 0.76 | no |
|  | 22 | 0.84 | Right caeca | 0.18 | no |
|  | 23 | 0.55 | Right caeca | 0.63 | no |
|  | 24 | 5.29 | Left caeca | 2.55 | no |
| **G4**  **(RecEtGRA9)** | 25 | 2.87 | Left caeca | 1.26 | no |
|  | 26 | 0.16 | Right caeca | 3.01 | **yes** |
|  | 27 | 0.06 | Right caeca | 8.07 | **yes** |
|  | 28 | 0.38 | Right caeca | 1.11 | no |
|  | 29 | 1.78 | Left caeca | 0.61 | no |
|  | 30 | 5.72 | Left caeca | 2.77 | no |
|  | 31 | 2.32 | Left caeca | 0.94 | no |
|  | 32 | 0.09 | Right caeca | 5.68 | **yes** |
| **G5**  **(recEtAMA1)** | 33 | 0.25 | Right caeca | 1.88 | no |
|  | 34 | 1.66 | Left caeca | 0.53 | no |
|  | 35 | 0.57 | Right caeca | 0.59 | no |
|  | 36 | 0.12 | Right caeca | 4.00 | **yes** |
|  | 37 | 0.02 | Right caeca | 22.97 | **yes** |
|  | 38 | 0.61 | Right caeca | 0.52 | no |
|  | 39 | 0.26 | Right caeca | 1.77 | no |
| **G6**  **(E. tenella**  **oocysts)** | 41 | 4.94 | Left caeca | 2.37 | no |
|  | 42 | 0.80 | Right caeca | 0.22 | no |
|  | 43 | x | Right caeca | - | no |
|  | 44 | 16.59 | Left caeca | 8.26 | **yes** |
|  | 45 | 0.63 | Right caeca | 0.47 | no |
|  | 46 | 2.33 | Left caeca | 0.95 | no |

**The ratio was determined by dividing the number of parasite genome copies found on the left caecum by those on the right caecum.*

Table VIII- Comparative analysis of parasite replication means among challenged groups.

| Group | Left caeca PRM* (SD) | Right caeca PRM (SD) | Predominant |
| --- | --- | --- | --- |
| G2 | 0.002419 (0.002922) | 0.003319 (0.003005) | Right |
| G3 | 0.001034 (0.001564) | 0.001903 (0.004293) | Right |
| G4 | 0.0008368 (0.001070) | 0.0005008 (0.0004268) | Left |
| G5 | 0.001826 (0.003844) | 0.003035 (0.006728) | Right |
| G6 | 3.970e-006 (5.172e-006) | 4.003e-006 (6.493e-006) | Right |

**PRM: parasite replication means. SD: standard deviation.*

BLAKE, D. P., QIN, Z., CAI, J. & SMITH, A. L. 2008. Development and validation of real-time polymerase chain reaction assays specific to four species of Eimeria. *Avian Pathology,* 37**,** 89-94.

CLARK, E. L., MACDONALD, S. E., THENMOZHI, V., KUNDU, K., GARG, R., KUMAR, S., AYOADE, S., FORNACE, K. M., JATAU, I. D. & MOFTAH, A. 2016. Cryptic Eimeria genotypes are common across the southern but not northern hemisphere. *International journal for parasitology,* 46**,** 537-544.

KOLASKAR, A. S. & TONGAONKAR, P. C. 1990. A semi-empirical method for prediction of antigenic determinants on protein antigens. *FEBS Lett,* 276**,** 172-4.

KYTE, J. & DOOLITTLE, R. F. 1982. A simple method for displaying the hydropathic character of a protein. *Journal of Molecular Biology,* 157**,** 105-32.

NOLAN, M. J., TOMLEY, F. M., KAISER, P. & BLAKE, D. P. 2015. Quantitative real-time PCR (qPCR) for Eimeria tenella replication-Implications for experimental refinement and animal welfare. *Parasitology International,* 64**,** 464-70.
